# Reversible guest-induced gate-opening with multiplex spin crossover responses in two-dimensional Hofmann clathrates†

**DOI:** 10.1039/d0sc04246c

**Published:** 2020-09-22

**Authors:** Rubén Turo-Cortés, Carlos Bartual-Murgui, Javier Castells-Gil, M. Carmen Muñoz, Carlos Martí-Gastaldo, José Antonio Real

**Affiliations:** Departamento de Química Inorgánica, Instituto de Ciencia Molecular (ICMol), Universidad de Valencia Valencia Spain carlos.bartual@uv.es jose.a.real@uv.es; Departamento de Física Aplicada, Universitat Politècnica de València Camino de Vera s/n E-46022 Valencia Spain

## Abstract

Spin crossover (SCO) compounds are very attractive types of switchable materials due to their potential applications in memory devices, actuators or chemical sensors. Rational chemical tailoring of these switchable compounds is key for achieving new functionalities in synergy with the spin state change. However, the lack of precise structural information required to understand the chemical principles that control the SCO response with external stimuli may eventually hinder further development of spin switching-based applications. In this work, the functionalization with an amine group in the two-dimensional (2D) SCO compound {Fe(5-NH_2_Pym)_2_[M^II^(CN)_4_]} (**1M**, 5-NH_2_Pym = 5-aminopyrimidine, M^II^ = Pt (**1Pt**), Pd (**1Pd**)) confers versatile host–guest chemistry and structural flexibility to the framework primarily driven by the generation of extensive H-bond interactions. Solvent free **1M** species reversibly adsorb small protic molecules such as water, methanol or ethanol yielding the **1M·H2O**, **1M·0.5MeOH** or **1M·xEtOH** (*x* = 0.25–0.40) solvated derivatives. Our results demonstrate that the reversible structural rearrangements accompanying these adsorption/desorption processes (**1M** ↔ **1M·guest**) follow a gate-opening mechanism whose kinetics depend not only on the nature of the guest molecule and that of the host framework (**1Pt** or **1Pd**) but also on their reciprocal interactions. In addition, a predictable and reversible guest-induced SCO modulation has been observed and accurately correlated with the associated crystallographic transformations monitored in detail by single crystal X-ray diffraction.

## Abstract 2

Layered Hofmann-type iron(ii) coordination polymers functionalised with 5-aminopyrimidine ligands show gate-opening driven guest-exchange accompanied by drastic structural and spin-crossover modulations.

## Graphical abstract

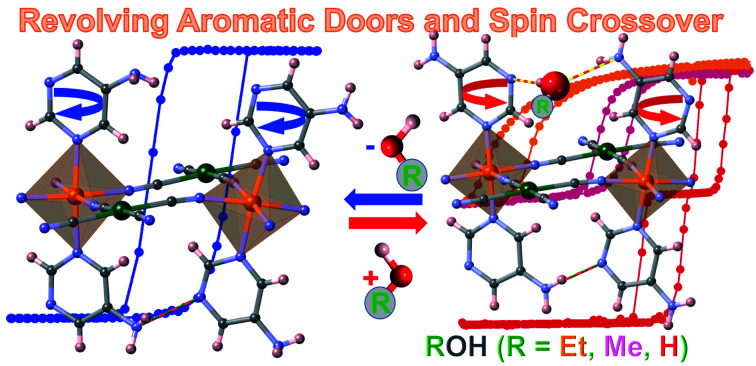

## Introduction

Hexa-coordinated iron(ii) spin crossover (SCO) complexes are a singular class of materials featuring a labile and reversible electronic configuration change between the diamagnetic low-spin state [t^6^_2g_e^0^_g_, *S* = 0] (LS) and the paramagnetic high spin state [t^4^_2g_e^2^_g_, *S* = 2] (HS).^1^ LS ↔ HS switching is an entropy-driven phenomenon which can be induced by a panoply of stimuli, *i.e.* a gradient of temperature^2^ and/or pressure,^3^ light irradiation,^4^ application of an electric field^5^ or even through interaction with analytes,^6^ thereby effecting changes in the magnetic, electric, optical, mechanical and structural properties of the material. In the solid state, the profile of the spin-state switch depends on the elastic coupling (cooperativity) between active SCO Fe^II^ centres. Gradual SCO spreading over a wide range of temperatures occurs when the elastic coupling is weak. On the contrary, strong elastic coupling favours sharp abrupt first order spin transitions which, in special cases, are accompanied by hysteretic behaviour conferring to the material bistability (memory effect). Materials exhibiting bistable SCO behaviour have attracted much interest because of their potential applications in memory devices, actuators or chemical sensors.^7^

In recent years, many studies have been devoted to the chemical design of SCO compounds in order to incorporate new functionalities acting in synergy with the purely thermal driven spin-state switching. These added properties include fluorescence,^8^ electrical conductivity^9^ or porosity,^10^ among others. Indeed, combination of SCO and porosity has been one of the most exploited routes for achieving multifunctionality in part due to its high potential in molecular sensor applications. The first examples showing coexistence of both properties were the doubly-interpenetrated compounds with general formula [FeL_2_(SCN)_2_]·Solv [L = 1,2-di-(4-pyridy1)-ethylene (tvp)^11^ or *trans*-4-4′azopyridine (azpy)^12^]. These compounds display SCO properties which depend on the included solvent molecules. Later, Hofmann-type coordination polymers (HCPs) formulated {Fe(L)_y_[M(CN)_x_]} {including non-interpenetrated [*x* = 4; L = monodentate (*y* = 2, 2D) or bis-monodentate (*y* = 1, 3D) ligand; M = Pd, Pt, Ni] or interpenetrated [*x* = 2, L = monodentate (*y* = 2, 2D) or bis-monodentate (*y* = 1, 3D) ligand; M = Ag, Au] compounds}^6*a*,13^ gained increasing interest due to their demonstrated structural versatility and the possibility of being processed as thin-films or nano-objects.^14^ The intrinsic structural porosity offered by this family of compounds has resulted in numerous reports studying synergies between SCO and host–guest chemistry.^15^ Overall, modulation of the SCO through guest adsorption can be explained by steric and/or electronic effects. The first factor involves stabilization of the HS state by the guest due to the hindering of the framework contraction associated with the HS-to-LS process.^16^ The second factor entails changes in the ligand field strength around the Fe^II^ centre *via* host–guest interactions with the coordinated ligands.^17^

A suitable synthetic strategy for inducing guest inclusion-SCO synergies is the use of asymmetric ligands with hydrophilic functional groups. This type of ligands promotes intermolecular interactions leading to lattice asymmetries which originate inter-sheet cavities where the guest molecules are located. This structural model was exploited using a series of asymmetric triazole-type ligands substituted with various chemical groups leading, in all cases, to multi-stable SCO compounds.^18^ Similar results were also obtained for pyridine donor asymmetric ligands.^19^ Overall, the shape of the SCO curves in these systems depends not only on the selected pillaring ligand but also on the amount and nature of the adsorbed guest molecules. For example, the SCO of compound {Fe(bztrz)_2_[Pd^II^(CN)_4_]}·G exhibits one, two or three steps when G = (H_2_O,EtOH), 3H_2_O or ∼2H_2_O, respectively, demonstrating that the elastic frustration which gives rise to multi-stability can be modulated by guest exchange.^18*e*^ Unfortunately, with very rare exceptions,^18*g*^ crystallinity of these 2D systems is partially or completely lost after total desorption of guest molecules preventing the evaluation of the involved structural modifications and their implication on the associated SCO changes. Moreover, the vanishing of crystallinity may also limit the accuracy of the structural characterization in a subsequent guest-dependent SCO study. The establishment of a dense network of host–host and host–guest intermolecular interactions (H-bonds, π–π stacking…) may be critical to overcome this limitation.

**Scheme 1:**
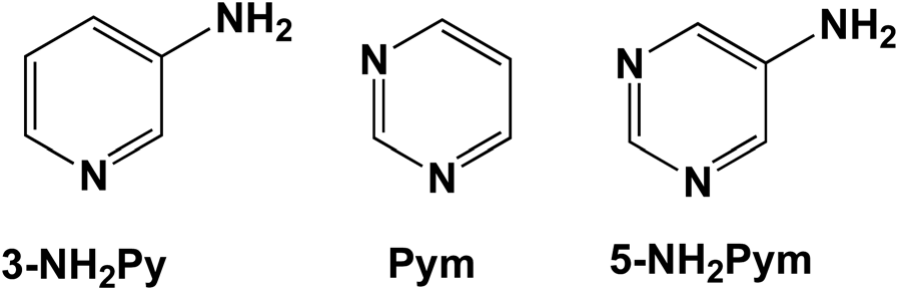
Related axial ligands used for the synthesis of new 2D Hofmann-type SCO coordination polymers (see text).

Recently, the analogous 2D compounds {Fe(3-NH_2_Py)_2_[M(CN)_4_]} and {Fe(Pym)_2_[M(CN)_4_]}·*x*H_2_O [3-NH_2_Py = 3-aminopyridine;^20^ Pym = pyrimidine^21^ (Scheme 1); M = Pt, Pd, Ni] were reported. The former displays host–host CH⋯N(amino) H-bonding interactions and hysteretic SCO for all the investigated derivatives, although no host–guest properties were described. In contrast, the latter exhibits guest-dependent cooperative spin transitions attributed to the H-bonds established between the non-coordinated nitrogen of the pyrimidine and the guest water molecules. However, the lack of detailed structural data after dehydration prevented the investigation of further precise magneto–structural correlations. In this work, the use of 5-aminopyrimidine (5-NH_2_Pym, Scheme 1) ligand has led to 2D HCPs compounds {Fe(5-NH_2_Pym)_2_[M^II^(CN)_4_]}·H_2_O [M^II^ = Pt (**1Pt·H2O**) or Pd (**1Pd·H2O**)]. The combination of a donor amino group and an acceptor non-coordinated nitrogen in the 5-NH_2_Pym axial ligand enables the coupling of contiguous [FeN_6_] octahedrons, belonging to the same layer, through a robust network of H-bond interactions which is additionally reinforced by the inclusion of protic guest molecules. Indeed, this stiff H-bond network seems to be key for the persistence of crystallinity upon the loss of water molecules that gives rise to unsolvated derivatives **1Pt** or **1Pd**. These guest-free compounds are prone to re-adsorb water or other small molecules as methanol or ethanol also following reversible single-crystal-to-single-crystal (SCSC) transformations. This has enabled us to establish precise correlations between the wide variety of SCO behaviours presented by this family of compounds and the structural transformations upon guest exchange (**1M** ↔ **1M·guest**). Interestingly, in agreement with the adsorption/desorption isotherm measurements, these crystallographic transformations follow a gate-opening mechanism which represents an unprecedented structural feature in combination with switchable 2D HCPs.

## Results

### Synthesis, structure and SCO properties of **1Pt·H2O** and **1Pd·H2O**

Single crystals of **1Pt·H2O** and **1Pd·H2O** were prepared by slow liquid-to-liquid diffusion methods from Fe(BF_4_)_2_·6H_2_O, K_2_[M^II^(CN)_4_] (M^II^ = Pt^II^ or Pd^II^) and 5-aminopyrimidine (5-NH_2_Pym) aqueous solutions separated by a water interphase in a double-H shaped tube (see experimental section in ESI†). Yellow thin plate-shaped single crystals were obtained in good yields (*ca.* 60–70%) after 4 weeks.

**Fig. 1:**
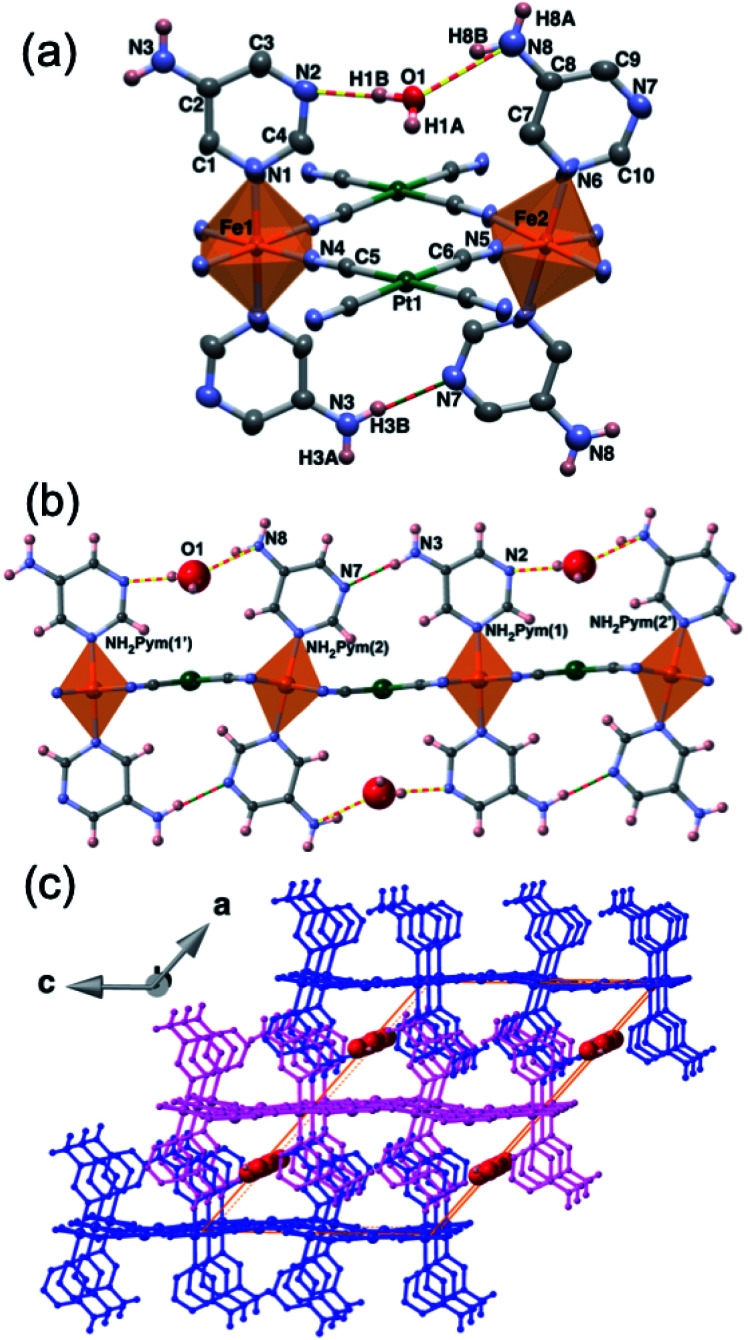
(a) ORTEP view of the asymmetric unit of **1Pt·H2O** at 260 K (isostructural to **1Pd·H2O**) showing 50% probability displacement ellipsoids (aromatic hydrogen atoms are omitted for clarity) and (b) view along the (010) direction of a **1M·H2O** (M = Pt, Pd) layer (discontinuous yellow-red and red-green lines represent host–guest and host–host H-bonds, respectively). (c) 3D supramolecular fragment of **1M·H2O** (M = Pt, Pd) displaying three successive pillared layers (distinguished in pink and blue). Red spheres represent the trapped water molecules within the 1D channels.

Compounds **1Pt·H2O** and **1Pd·H2O** are isomorphous and crystallize in the monoclinic *C*2/*m* space group. The asymmetric unit is constituted by two non-equivalent [Fe^II^1N_6_] and [Fe^II^2N_6_] pseudo-octahedral centres connected through their equatorial coordination sites by two equivalent [M^II^(CN)_4_]^2−^ units (M^II^ = Pt or Pd) (Fig. 1a), thereby defining cyano-bridged bimetallic Fe^II^–M^II^ layers (Fig. 1b). The axial positions of each Fe^II^ ion are coordinated by two equivalent terminal 5-NH_2_Pym ligands through one of its two heterocyclic N atoms. At 260 K, the average [Fe1N_6_]/[Fe2N_6_] bond lengths, 2.159 Å/2.164 Å for **1Pt·H2O** and 2.168 Å/2.171 Å for **1Pd·H2O**, are consistent with a fully populated HS state. Within a given Fe^II^ centre, the apical 5-NH_2_Pym ligands [hereafter labelled as 5-NH_2_Pym(1) (coordinated to Fe^II^1) and 5-NH_2_Pym(2) (coordinated to Fe^II^2)] are disposed in such a way that they adopt a *trans* conformation with respect to the orientation of the amino substituent. This enables the formation of two types of H-bonds within each layer. One, a single H-bond between the N3 atom of the amino group of 5-NH_2_Pym(1) and the uncoordinated N7 heteroatom of the adjacent 5-NH_2_Pym(2) ligand. The other, a double H-bond involving the adjacent N2 and N8 atoms belonging to the heteroatom of 5-NH_2_Pym(1) and the amino group of 5-NH_2_Pym(2′), respectively, mediated by the interaction with the guest water molecule (Fig. 1a and b). The trapped water molecule is located within two discrete equivalent positions modelled with an occupancy of 0.5 (0.4 in the case of Pt), hence the structure contains 1 (0.8 for Pt) molecule of water per Fe^II^ ion in good agreement with the TGA studies (Fig. S1a and b†). The intralayer H-bond interactions define an array of parallel linear chains running along the (001) direction. As a result, the bimetallic layers are slightly corrugated (Fig. 1b) being the angles defined by the equatorial planes of the coordination Fe1–Fe2/Fe1–Pt(Pd)/Fe2–Pt(Pd) centres in the interval 14.8–15°/1.3–4.0°/16.2–18.8°, respectively. The layers are pillared in such a way that the apical 5-NH_2_Pym ligands are interdigitated defining weak π–π interactions (Fig. S2a†) and the M^II^ centres of one layer are on top the centre of the [Fe_2_M^II^_2_] windows of the adjacent layers (Fig. S2b†). The packing of the layers generates 1D channels where the water molecules are located (Fig. 1c). The interlayer distance based on the average plane defined by the Fe1–Fe2–Pt/Pd atoms is 8.17 and 8.14 Å for **1Pt·H2O** and **1Pd·H2O**, respectively. Host–host and host–guest H-bond interactions found for **1Pt·H2O** and **1Pd·H2O** and for the rest of studied solvates are gathered in Table 1.

**Table 1:** Selected H-bond interactions (in Å) found for **1M**, **1M·H2O**, **1M·0.5MeOH** and **1Pt·0.4EtOH** at 260 K

Interaction	**1Pt**	**1Pd**	**1Pt·H2O**	**1Pd·H2O**	**1Pt·0.5MeOH**	**1Pd·0.5MeOH**	**1Pt·0.4EtOH**
**Host–host**							
N6(amino)⋯N7(het.)	3.023	2.973	—	—	—	—	—
N3(amino)⋯N7(het.)	—	—	3.106	3.075	—	—	3.053
N7(amino)⋯N3(het.)	—	—	—	—	2.979	3.039	—
N11(amino)⋯N13(het.)			—	—	3.015	3.042	—

**Host–guest**							
O1(guest)⋯N2(het.)	—	—	2.885	2.842	—	—	2.795
O1(guest)⋯N8(amino)	—	—	3.053	3.035	—	—	2.963
O1(MeOH)⋯N2(het.)	—	—	—	—	2.724	2.771	—
O2(MeOH)⋯N10(het.)	—	—	—	—	2.805	2.781	—
O1(MeOH)⋯N8(amino)	—	—	—	—	2.900	2.914	—
O2(MeOH)⋯N14(amino)	—	—	—	—	2.979	2.908	—

**Fig. 2:**
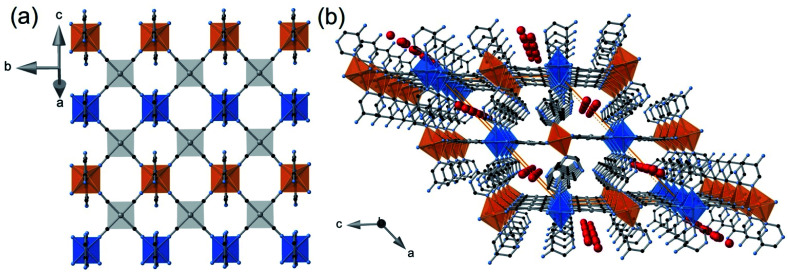
Perspective views of **1Pt·H2O** at 187 K (structurally equivalent to **1Pd·H2O** at 180 K) displaying (a) a bimetallic layer in the ⋯HS–LS⋯ ordered intermediate state representing in blue the LS Fe1 sites and in orange the HS Fe2 sites and (b) three stacked layers showing the planes containing the HS or LS sites (hydrogen atoms are omitted for clarity).

Upon cooling to 187 K (180 K for **1Pd·H2O**), the crystals become orange suggesting the occurrence of a HS-to-LS state change. The system retains the *C*2/*m* space group and the overall structure does not change significantly with respect to that at 260 K. However, whereas the average [Fe1N_6_] bond length decreases by 0.194 Å for **1Pt·H2O** and **1Pd·H2O**, that of [Fe2N_6_] remains barely unaltered for both **1Pt·H2O** (2.165 Å *vs.* 2.139 Å) and **1Pd·H2O** (2.171 Å *vs.* 2.156 Å). These values reveal that whereas Fe1 centres undergo a complete HS-to-LS transition, those of Fe2 remain in the HS configuration. This defines a ⋯HS–LS⋯ ordered state within the linear H-bond chains mentioned above (Fig. 2a) that in turn results in an infinite ordered succession of HS and LS planes running along (001) (Fig. 2b). Further cooling to 120 K induces a complete spin transition as indicated by the Fe1–N/Fe2–N average distances of 1.948/1.955 and 1.961/1.963 Å for **1Pt·H2O** and **1Pd·H2O**, respectively. These structural data are in perfect agreement with the magnetic measurements (*vide infra*). The HS → LS process is accompanied by a contraction of the interlayer distance by 0.3 Å and 0.2 Å for **1Pt·H2O** and **1Pd·H2O**, respectively. Furthermore, the angles defined by the equatorial planes of Fe1–Fe2/Fe1–Pt(Pd)/Fe2–Pt(Pd) decrease by 0.7°/1.2°/2.0° when moving from the HS to the LS state for **1Pt·H2O** and **1Pd·H2O**, consequently the undulation of the {Fe[M(CN)_4_]}_*n*_ planes is slightly less pronounced. The main crystallographic parameters of **1Pt·H2O** and **1Pd·H2O** are gathered in Tables S1 and S2,† respectively.

**Fig. 3:**
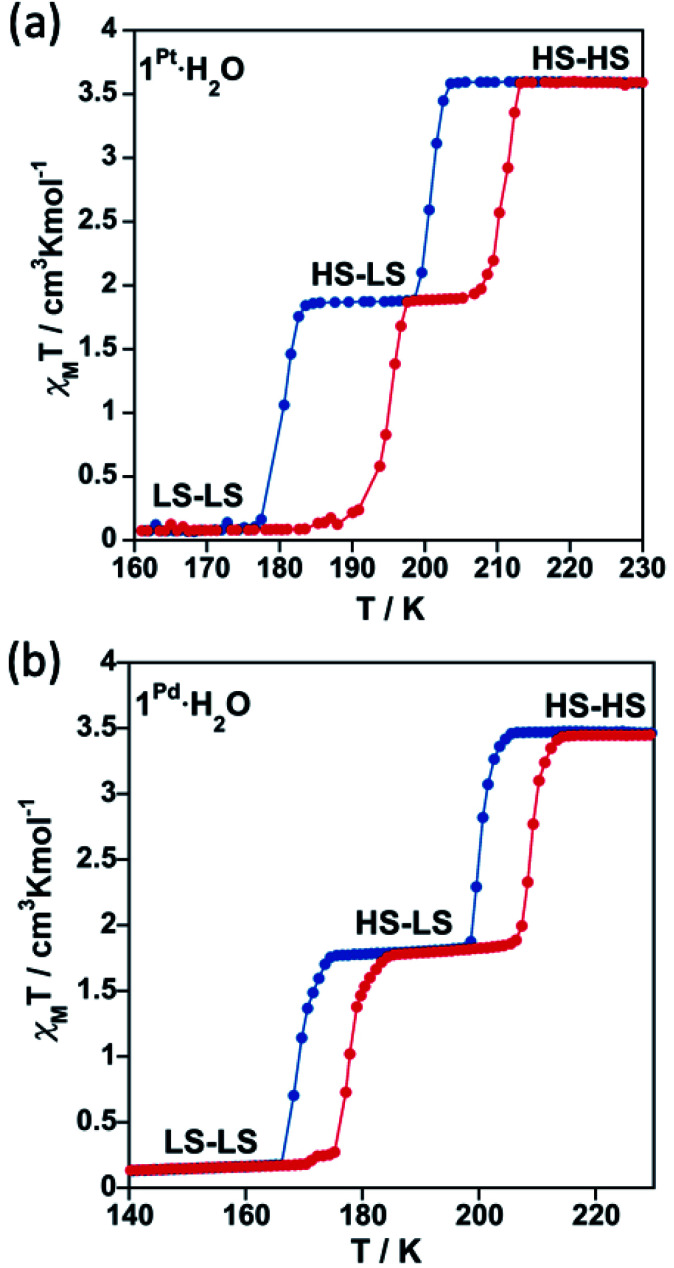
SCO behaviour expressed as *χ*_M_*T vs. T* plots recorded at 2 K min^−1^ for (a) **1Pt·H2O** and (b) **1Pd·H2O**. Cooling and heating modes are highlighted in blue and red, respectively.

Fig. 3 shows the magnetic properties in the form of *χ*_M_*T versus T* plots (*χ*_M_ is the molar magnetic susceptibility and *T* is the temperature) for **1Pt·H2O** and **1Pd·H2O**. Samples of **1Pt·H2O** and **1Pd·H2O** and, consequently, those of their corresponding derivatives (*vide infra*) were constituted exclusively of single crystals which, according to the elemental analysis and powder X-ray diffraction studies (see experimental section and Fig. S3 in ESI†), corresponded to a single phase. At 290 K, the *χ*_M_*T* value of the as-synthesized crystals of **1Pt·H2O** and **1Pd·H2O** was found to be about 3.5 cm^3^ K mol^−1^, which is consistent with the Fe^II^ ion in the HS state. When cooling at 2 K min^−1^, this value remains constant until it drops abruptly just below *ca.* 205 K in two marked steps, involving each one 50% of a complete HS-to-LS transformation. The average critical temperatures *T*_c_ (calculated as *T*_c_ = *T*^↑^_c*i*_ + *T*^↓^_c*i*_/2 where *T*^↑^_c*i*_ and *T*^↓^_c*i*_ (*i* = 1, 2) are the transition temperatures in the respective heating and cooling modes) are *T*_c1_ = 205.5 (204) K and *T*_c2_ = 187.5 (173.5) K for **1Pt·H2O** (**1Pd·H2O**) within the first and second step, respectively, and define hysteresis loops (Δ*T*_c_ = *T*^↑^_c*i*_ − *T*^↓^_c*i*_) of Δ*T*_c1_ = 9 (8) K and Δ*T*_c2_ = 15 (9) K for **1Pt·H2O** (**1Pd·H2O**). Hence, the SCO curves of **1Pt·H2O** and **1Pd·H2O** define a plateau in the temperature range 184–205 K and 175–205 K, respectively, where, accordingly to the structural data, a HS–LS mixed state is present.

### Guest exchange properties (**1M·H2O** ↔ **1M** ↔ **1M·guest**)

**Scheme 2:**
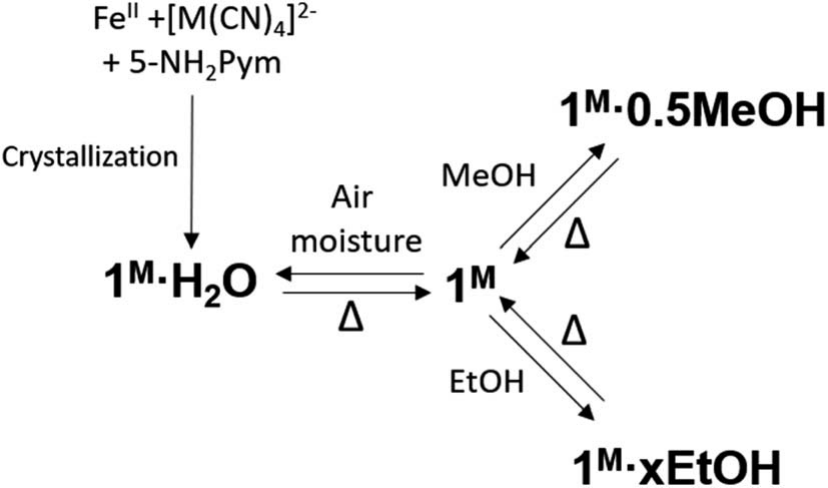
Scheme of the main chemical transformations reported in this work.

**Fig. 4:**
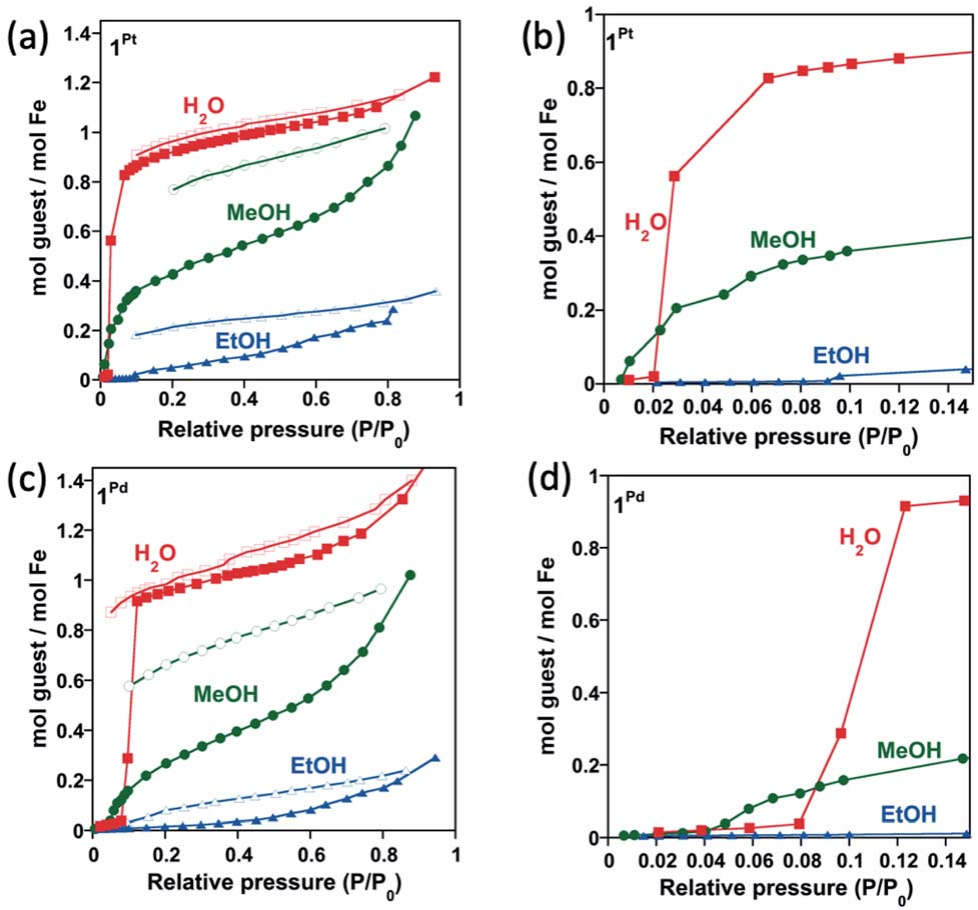
Water, methanol and ethanol isothermal adsorption (full points) and desorption (open points) curves for **1Pt** and **1Pd** in (a and c) full scale and (b and d) the corresponding enlarged plots at low pressures range (desorption curves are omitted in the low pressures centred curves).

Based on the thermogravimetric analysis (TGA) (Fig. S1a and b†), **1Pt** and **1Pd** solvent-free single crystals were prepared by removing the included water molecule from the corresponding **1Pt·H2O** and **1Pd·H2O** counterparts through thermal treatment at 400 K for 30 minutes. The anhydrous derivatives spontaneously uptake water from atmospheric moisture yielding the primitive hydrated **1Pt·H2O** and **1Pd·H2O** derivatives (see Scheme 2). To in depth analyse this behaviour, water adsorption isotherms were performed for **1Pt** and **1Pd**. These compounds do not show significant water adsorption below a value of relative pressure, *P*/*P*_0_, equal to 0.02 for **1Pt** and 0.08 for **1Pd** (see Fig. 4a and c and the corresponding enlarged plots in Fig. 4b and d). Above this threshold value, a sigmoidal adsorption profile takes place first rapidly reaching a covering value of 0.83 and 0.90 mol H_2_O/mol Fe for **1Pt** and **1Pd**, respectively, and then more gradually to reach a value of *ca.* 1 mol H_2_O/mol Fe at *P*/*P*_0_ = 0.6. This result suggests a gate-opening mechanism in which the adsorption process is accompanied by a drastic cooperative crystallographic transformation. Indeed, the resulting curve corresponds to a Type F-IV adsorption profile typical for flexible compounds exhibiting non-porous to porous structural transitions.^22^

In subsequent SCSC transformations, desorbed **1Pt** and **1Pd** crystals soaked in MeOH or EtOH for a period of 3 h afforded crystals of **1M·0.5MeOH** or **1M·xEtOH** (M = Pt, Pd, *x* = 0.25–0.4) (see Scheme 2). According to the corresponding TGA, a maximum of 0.5 molecules of methanol per Fe^II^ are trapped within the structure of **1M** whereas *ca.* 0.4 molecules of ethanol was included in **1Pt** (**1Pt·0.4EtOH**). However, only 0.25 molecules of ethanol were found in **1Pd** (**1Pd·0.25EtOH**) (Fig. S1c–f†). Given the non-porous nature of **1Pt** and **1Pd**, and due to the larger molecular volume of MeOH, and especially of EtOH, their adsorption isotherms reflect much higher steric hindrance for these guests than for H_2_O, thereby requiring higher *P*/*P*_o_ values to include amounts close to the ones found for the soaked crystals. Interestingly, the MeOH adsorption isotherm for **1Pd** also displays a clear sigmoidal shape with a threshold relative pressure of 0.04 *P*/*P*_0_ (see Fig. 4d) reflecting a Type F-III adsorption profile ascribed to a gradual non-porous to porous structural switching.^22^ Indeed, this threshold value is even lower than that of H_2_O and the same trend is observed for the Pt derivative (0.01 *P*/*P*_0_ for MeOH *vs.* 0.02 for water) (Fig. 4b). In addition, the desorption isotherm profiles for the MeOH and EtOH derivatives are significantly different to the adsorption ones defining a marked hysteretic behaviour. This fact contrasts with the water uptake whose adsorption/desorption profiles are very close each other. All these observations suggest that the host clathrates exhibit higher chemical affinity for MeOH than for H_2_O at very low guest pressures and, although relatively strong interactions seem to be stablished with EtOH, its higher molecular volume may prevent an efficient adsorption. Similar results describing lower adsorption pressures for MeOH and EtOH than for H_2_O in a non-SCO 2D framework have been reported and ascribed to the presence of hydrophobic interactions.^23^

Time-dependent thermogravimetic studies of **1M** under water, methanol or ethanol vapours are consistent with the results described above. With the aim of extracting the kinetic parameters associated with the adsorption processes, the quantity of adsorbed guest as a function of time was fitted to the Avrami equation^24^ (*α* = *A*(1 − exp{−*K*_av_*t*^*n*^})) (see Fig. S4 and Table S3†). As expected regarding the adsorption isotherms, the Pt derivative presents higher amounts of adsorbed guest (*A*) and higher adsorption kinetic constants (*K*_av_) than the Pd one. In addition, for a given activated derivative (**1Pt** or **1Pd**), the adsorption kinetic constants are higher for MeOH and EtOH than for water suggesting a stronger affinity by the host framework for the formers. However, the *n* parameter, which accounts for the cooperativity of the process, indicates that the adsorption event is more cooperative for water than for the alcohol molecules. Furthermore, for a given guest molecule, *n* is higher for the Pd than for the Pt derivative.

### Guest exchange-induced structural modifications

Single crystal X-ray diffraction measurements were performed in order to assess the structural modifications involved upon subsequent guest exchanges (**1M·H2O** ↔ **1M** ↔ **1M·guest**). Furthermore, each compound was measured at temperatures in which the different spin states (HS, LS and/or mixed ⋯HS–LS⋯) manifest according to the magnetic properties (*vide infra*). Crystals of **1Pt·H2O** and **1Pd·H2O** were *in situ* heated in the diffractometer at 400 K in order to remove the guest water molecule, thereby yielding the corresponding **1Pt** and **1Pd** dehydrated phases. The water-free structures of **1Pt** and **1Pd** were then successfully determined at 260 K (HS) and 120 K (LS). In a subsequent step, **1Pt** and **1Pd** were loaded with MeOH to give **1Pt·0.5MeOH** and **1Pd·0.5MeOH** and the crystallographic data of freshly prepared crystals collected at 260 K (HS–HS) and 100 K (HS–LS). The ethanol derivatives were prepared in a similar way. In particular, **1Pt·0.4EtOH** was analysed at 260 K (HS–HS) and 100 K (HS–LS). However, the low quantity of ethanol adsorbed by **1Pd** led to mixed crystallographic phases that prevented the proper resolution of the structure of **1Pd·0.25EtOH**. The main crystallographic parameters of the discussed structures are displayed in Tables S1, S2, S4 and S5.†

### Structure of **1Pt** and **1Pd**

**Fig. 5:**
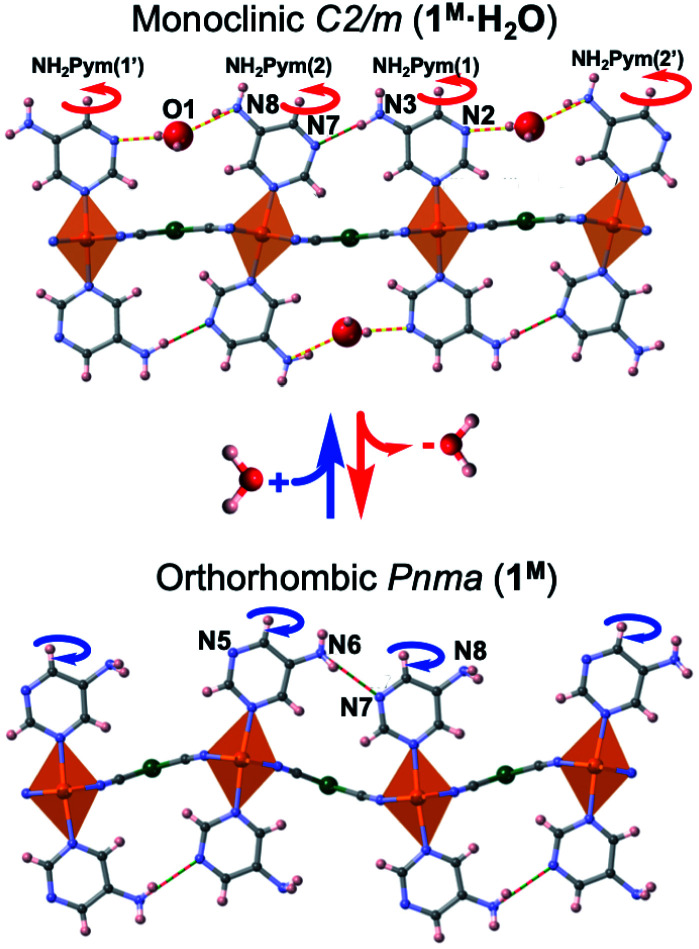
Views along the (010) direction of a bimetallic layer of **1M·H2O** (up) and **1M** (down) at 260 K (M = Pt, Pd). Red/blue curved arrows indicate the 180° rotation of the 5-NH_2_Pym ligands occurring along with the dehydration/hydration processes (discontinuous yellow-red and red-green lines represent host–guest and host–host H-bonds).

Removal of the included water molecule from **1M·H2O** (**1M·H2O** → **1M**) involves a crystallographic phase transformation from the monoclinic *C*2/*m* to the orthorhombic *Pnma* space group. Although the layered structure of **1M** is comparable to that of the hydrated monoclinic phase, the loss of water is accompanied by noticeable structural modifications (Fig. 5): (i) Only one type of Fe^II^ ion, axially coordinated by two crystallographically distinct 5-NH_2_Pym ligands, is now observed; (ii) one of the two axial 5-NH_2_Pym is rotated 180° in such a manner that they adopt a *cis* conformation with respect to the orientation of the amine groups; (iii) the void space generated upon desorption of water molecules is minimized by subtle re-accommodation of the bimetallic layers whose undulated corrugation is more noticeable and regular. Indeed, the equatorial plane around Fe1 defines an angle with the [M(CN)_4_]^2−^ plane of 12.14° (M = Pt) and 14.95° (M = Pd) in the LS state but increases considerably up to 24.67° (M = Pt) and 29.59° (M = Pd) in the HS; (iv) the absence of included water molecules prevents the formation of the H-bonding chains observed for **1Pt·H2O** and **1Pd·H2O**. Instead, only one intralayer H-bond interaction (N6⋯N7) is established between adjacent 5-NH_2_Pym ligands (Fig. 5 and Table 1).

At 260 K, the [FeN_6_] average bond length [2.161 and 2.158 Å for **1Pt** and **1Pd**, respectively] is consistent with 100% of the Fe^II^ ions in the HS state. When cooling to 120 K, the structures remain in the *Pnma* space group but the [FeN_6_] average bond length decreases to 1.950 Å for **1Pt** and 1.960 Å for **1Pd**, consistently with a complete HS-to-LS transition, also reflected by the observed yellow to red colour change. It is important to note that, according to the powder X-ray diffraction (Fig. S5†), the spontaneous readsorption of water from air moisture (**1M** → **1M·H2O**) shows complete structural reversibility recovering the original **1M·H2O** frameworks.

### Structure of **1Pt·0.5MeOH** and **1Pd·0.5MeOH**

The **1M** → **1M·0.5MeOH** adsorption process involves a crystallographic phase change from the orthorhombic *Pnma* to the orthorhombic *Pbcm* space group. Overall, **1Pt·0.5MeOH** and **1Pd·0.5MeOH** present the same general structure as their water counterparts (**1Pt·H2O** and **1Pd·H2O**) but the asymmetric unit is constituted, in addition to two different [Fe^II^N_6_] centres, of two inequivalent 5-NH_2_Pym per Fe^II^ and two distinct [M^II^(CN)_4_]^2−^ bridging units (Fig. S6†). As in **1Pt·H2O** and **1Pd·H2O**, the two 5-NH_2_Pym apical ligands coordinated to a given Fe^II^ centre display a *trans* conformation with respect to the amine group orientation revealing that 50% of the axial ligands undergoes a 180° rotation upon MeOH adsorption. Similarly to **1M·H2O**, the bimetallic layers are slightly corrugated as shown by the angles defined between consecutive equatorial planes of the metallic centres [Fe1–M1/Fe2–M1/Fe1–M2/Fe2–M2] being equal to [1.96° (2.27°)/13.24° (17.59°)/1.01° (0.25°)/16.22° (15.07°)] for **1Pt·0.5MeOH** and [1.43° (1.28°)/15.70° (14.15°)/1.25° (2.12°)/14.02° (17.56°)] for **1Pd·0.5MeOH** in the LS and (HS) states, respectively. This irregular corrugation seems also to be guided by the formation of host–host and host–guest H-bonds. The bimetallic layers are now stacked along the (010) direction generating an array of two inequivalent 1D interlayer channels, running along the (001) direction and delimited by the M^II^(1) or M^II^(2) centres, respectively. These channels host an average of *ca.* 0.5 molecules of MeOH per Fe^II^ (Fig. S7†) in good agreement with the thermogravimetric analysis (Fig. S1c and d†). The corresponding oxygen atom interacts *via* H-bonding with both the non-coordinated nitrogen heteroatom and the amino group of the apical 5-NH_2_Pym ligands (Table 1). The much narrower channels created between the bimetallic layers are blocked by the (Pym)NH_2_⋯N(Pym)NH_2_ hydrogen bonds.

At 260 K, the average Fe1–N/Fe2–N bond lengths (2.174 Å/2.166 Å and 2.175 Å/2.173 Å for **1Pt·0.5MeOH** and **1Pd·0.5MeOH**, respectively) are consistent with a fully populated HS state. When cooling down to 100 K, the crystal retains the orthorhombic *Pbcm* space group and undergoes a yellow-to-dark orange color change indicating the occurrence of a HS-to-LS change. Indeed, although the Fe1 site remains in the HS state (Fe1–N average distance = 2.174 Å (260 K) *vs.* 2.164 Å (100 K)/2.175 Å (260 K) *vs.* 2.154 Å (100 K) for **1Pt·0.5MeOH**/**1Pd·0.5MeOH**), Fe2 undergoes a complete switch to the LS state (Fe2–N average distance = 2.166 Å (260 K) *vs.* 1.984 Å (100 K)/2.173 Å (260 K) *vs.* 1.985 Å (100 K) for **1Pt·0.5MeOH**/**1Pd·0.5MeOH**) giving rise to an ordered ···HS–LS··· state. The packing of the layers becomes slightly denser during the SCO event as it is reflected by the decrease of the distance between the average planes, defined by the undulated cyano-bridged bimetallic layers, from 8.197 Å/8.097 Å to 7.997 Å/7.987 Å for **1Pt·0.5MeOH** and from 8.185 Å/8.138 Å to 8.070 Å/8.013 Å for **1Pt·0.5MeOH**. Powder X-ray diffraction measurements have confirmed the structural reversibility of the methanol adsorption since the resulting pattern of the solid upon heating (**1M·0.5MeOH** → **1M**) coincides with that initially registered for the dehydrated compound (**1M·H2O** → **1M**) (Fig. S8†).

### Structure of **1Pt·0.4EtOH**

The adsorption of ethanol in **1Pt** provokes a crystallographic phase transition from the orthorhombic *Pnma* to the monoclinic *I*2/*m* space group. The structure of **1Pt·0.4EtOH** is homologous to that of **1M·H2O** the main difference residing in the distinct nature of the included guests. Hence, in excellent accord with the TGA (Fig. S1e†), 0.4 molecules of ethanol are located within the 1D channels in such a way that they form hydrogen bonds with both the amino and the heterocyclic N atom of adjacent 5-NH_2_Pym ligands (Table 1).

At 260 K, the Fe1–N/Fe2–N average distances are 2.164/2.168 Å, thereby reflecting a fully populated HS state. When cooling to 100 K, these distances change to 2.112/1.987 Å revealing that whereas the *ca.* 70% of the Fe1 centres remain in the HS state, the Fe2 centres undergo a complete transition to the LS state. Table 2 gathers the Fe–N average distances and the octahedral distortion parameters at each temperature for all the studied structures.

Octahedral distortion parameters (*Θ* and *Σ*) at different temperatures and the associated Fe–N average distances for **1Pt·H2O** and **1Pd·H2O**. (*Θ* is defined as 
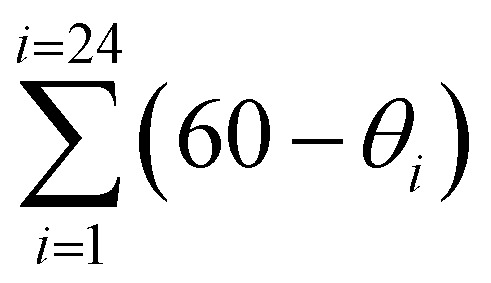
 being *θ*_*i*_ the angle generated by superposition of two opposite faces of the octahedron there are four pairs of such superposition with six *θ*_*i*_ values each one). *Σ* represents octahedron distortion defined as the sum of deviations from 90° of the 12 *cis* N–Fe–N angles in the coordination sphere 
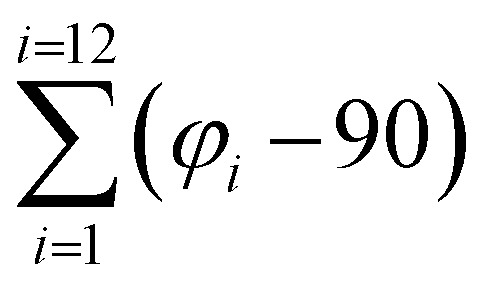

**Table:** 

Sample	**1Pt**		**1Pt·H2O**					
*T* (K)	260	120	260		187		100	
Spin state	HS	LS	Fe1(HS)	Fe2(HS)	Fe1(LS)	Fe2(HS)	Fe1(LS)	Fe2(LS)
Fe–N (Å)	2.161	1.950	2.159	2.164	1.965	2.140	1.948	1.955
*Θ*/*Σ*	39/17.9	20.1/8.4	22/11.6	39/22.8	21/12.0	43/29.6	17/14.0	30/20.4

**Table:** 

Sample	**1Pd**		**1Pd·H2O**					
*T* (K)	260	120	260		180		100	
Spin state	HS	LS	Fe1(HS)	Fe2(HS)	Fe1(LS)	Fe2(HS)	Fe1(LS)	Fe2(LS)
Fe–N (Å)	2.158	1.960	2.168	2.174	1.987	2.156	1.961	1.963
*Θ*/*Σ*	52/23	18.7/9.2	16/4.8	28/14.8	19/9.2	33/25.2	14/5.6	29/13.2

**Table:** 

Sample	**1Pt·0.5MeOH**				**1Pd·0.5MeOH**			
T (K)	260		100		260		100	
	Fe1(HS)	Fe2(HS)	Fe1(HS)	Fe2(LS)	Fe1(HS)	Fe2(HS)	Fe1(HS)	Fe2(LS)
Fe–N (Å)	2.174	2.166	2.164	1.984	2.174	2.173	2.155	1.986
*Θ*/*Σ*	29/4.7	28/19.7	37/17.6	26/9.7	32/8.4	28/22.4	32/18.0	25/11.9

**Table:** 

Sample	**1Pt·0.4EtOH**			
T (K)	260		100	
	Fe1(HS)	Fe2(HS)	Fe1(HS)	Fe2(LS)
Fe–N (Å)	2.164	2.166	2.112	1.986
*Θ*/*Σ*	17/7.2	36/22.8	34/16.4	22/15.6

### Guest-dependent SCO properties of **1M**

**Fig. 6:**
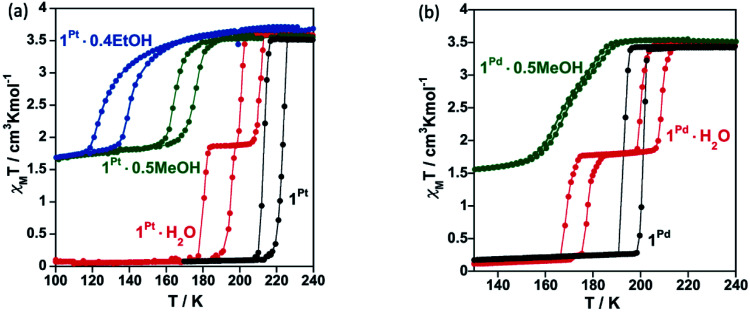
SCO behaviour for (a) **1Pt**, **1Pt·0.5MeOH** and **1Pt·0.4EtOH** and (b) **1Pd** and **1Pd·0.5MeOH** (scan rate: 2 K min^−1^). Spin transitions curves of hydrated compounds (**1Pt·H2O** and **1Pd·H2O**) are also displayed for comparison.

Compounds **1Pt·H2O** and **1Pd·H2O** were dehydrated *in situ* in the SQUID chamber at 400 K for one hour to afford **1Pt** and **1Pd** and their *χ*_M_*T vs. T* curves subsequently recorded (see Fig. 6a and b, respectively). The resulting spin transitions remain abrupt and complete although they occur in a single step. In the case of the Pt derivative the critical temperature increases (*T*_c_ = 218 K, Δ*T*_c_ = 10 K) with respect to those of the hydrated counterpart. In contrast, for **1Pd**, the critical temperature (*T*_c_ = 196.5, Δ*T*_c_ = 9 K) lie roughly in between the two hysteresis loops displayed by **1Pd·H2O**. Importantly, as mentioned above, **1M** recover the water molecule when exposed to air moisture yielding the initial **1M·H2O** compounds and showing full reversibility of the SCO properties (Fig. S9†).

The SCO properties of **1Pt·0.5MeOH**/**1Pt·0.4EtOH** and **1Pd·0.5MeOH** are displayed in Fig. 6a and b, respectively. The adsorption of MeOH induces a significant decrease of the SCO temperatures and whereas the transition remains cooperative displaying a one-step hysteretic curve (Δ*T*_c_ = 10 K) with *T*_c_ = 170 K for **1Pt·0.5MeOH**, it exhibits a very subtle two-step behaviour centred at similar temperatures (*T*_c_ = 170.5 K) with a narrow hysteresis (Δ*T*_c_ = 3 K) for **1Pd·0.5MeOH**. The inclusion of ethanol in **1Pt** (compound **1Pt·0.4EtOH**) induces an even further decrease of the spin crossover temperatures than the methanol does (*T*_c_ = 131.5 K) while conserving a 13 K hysteresis wide. The *χ*_M_*T* values recorded at 100 K (1.70/1.43/1.63 cm^3^ K mol^−1^ for **1Pt·0.5MeOH**/**1Pd·0.5MeOH**/**1Pt·0.4EtOH**) indicate, in good agreement with the structural data, that the presence of MeOH or EtOH blocks *ca.* 49/41/46% of the Fe^II^ ions in the HS state. Despite further cooling to 50 K no additional spin transition events were observed for these compounds (Fig. S10†). In contrast, the *χ*_M_*T vs. T* curve of **1Pd·0.25EtOH** displays a more complete two-step SCO behaviour (Fig. S11a†). The first step is characterized by a cooperative transition with *T*_c1_ = 191 K (Δ*T*_c1_ = 10 K) whereas the second one shows a gradual transition with *T*_c2_ = 146.5 K and (Δ*T*_c2_ = 9 K). Among the *ca.* 79% of the Fe^II^ centres that are SCO-active in **1Pd·0.25EtOH**, *ca.* 47% undergo the spin transition in the first step and *ca.* 32% in the second step. As shown by TGA (Fig. S1e, f and S11b†), the differences in the SCO properties between **1Pt·0.4EtOH** and **1Pd·0.25EtOH** are likely assignable to the lower effective quantity of ethanol present in the latter (0.4 *vs.* 0.25, respectively).

The SCO properties of **1Pt**, **1Pd**, **1Pt·H2O**, **1Pd·H2O**, **1Pt·0.5MeOH** and **1Pd·0.5MeOH** were also monitored through differential scanning calorimetry (DSC) at 10 K min^−1^. **1Pt·0.4EtOH** and **1Pd·0.25EtOH** were not analysed by this technique since their corresponding spin transitions are out of the temperature window of our calorimeter. As depicted in Fig. S12,† DSC measurements reproduce very well the SCO behaviour observed for the different samples in the magnetic studies. For example, calorimetric curves of dehydrated (**1M**) and hydrated (**1M·H2O**) compounds show one and two singularities during both the heating and cooling modes confirming single and double stepped SCO behaviours, respectively. In addition, the Δ*H*/Δ*S* (kJ mol^−1^/J K^−1^ mol^−1^) parameters are 14.63/74.86, 18.06/82.32, 17.49/88.14 and 16.49/86.52 for **1Pt**, **1Pd**, **1Pt·H2O** and **1Pd·H2O**, respectively, in good agreement with the values typically displayed by Hofmann-like Fe^II^ compounds featuring cooperative and complete SCO behaviours.^4*a*^ In contrast, **1Pt·0.5MeOH** and **1Pd·0.5MeOH** solvates present Δ*H*/Δ*S* (kJ mol^−1^/J K^−1^ mol^−1^) values of 7.64/40.09 and 7.10/41.40 consistent with a *ca.* 50% blocking of the spin transition as detected in the corresponding magnetic measurements.

## Discussion

The adsorption isotherms indicate different sorption capabilities for **1Pt** and **1Pd** derivatives. Indeed, under the same conditions, **1Pt** adsorbs a higher amount of guest and with faster kinetics than **1Pd** (see Fig. 4, S4 and Table S3†). It is worth mentioning that **1Pt** and **1Pd** desolvated forms do not present intrinsic porosity and, therefore, the uptake process occurs concomitantly to noticeable structural modifications which enable the entry of guests giving place to a gate-opening adsorption mechanism. Although related phenomena have been reported for 0D^25^ and 1D^26^ Co(ii) SCO systems, the lack of precise structural data associated to the uptake processes prevented direct information about its origin. Kitagawa et *al.* showed a gate-opening effect on 2D nanometric thin films of {Fe(Pyridine)_2_[Pt(CN)_4_]} revealing that, upon adsorption, the guest molecules are hosted by inducing separation between the stacked 2D layers.^27^ In contrast to the latter related example, our results here described disclose that the mechanism of structural reaccommodation upon guest sorption/desorption involves a 180° rotation of 50% of the 5-NH_2_Pym axial ligands which seems to facilitate the diffusion of the guest throughout the channels. Similar “revolving door” effect has been observed in discrete^28^ and 1D^29^ SCO systems. Another relevant structural change accompanying the guest uptake involves breaking the N6⋯N7 H-bond operating between the amino group and the non-coordinated nitrogen of adjacent 5-NH_2_Pym ligands (Fig. 5). Once the energy barrier of this rupture process is overcome the adsorption occurs in a cooperative one-step fashion revealing the gate-opening nature. Thus, the rupture of this interaction may determine the adsorption profile for each derivative. As a consequence, the slower adsorption regime of **1Pd** with respect to **1Pt** may be attributed to the stronger N6⋯N7 H-bond interaction of the former (Table 1). Furthermore, the accessible pore volumes calculated with PLATON for the corresponding **1M·guest** structures (Table S6†) are slightly higher for Pt than for Pd derivatives, which probably facilitates the uptake and dissemination of guest molecules within the former.

**Fig. 7:**
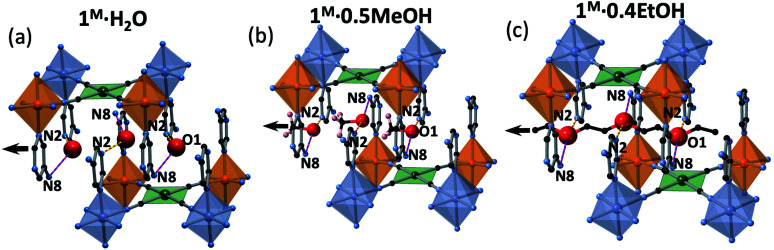
View of a fragment of two consecutive layers emphasising the specific sites occupied by the guests, (a) H_2_O, (b) MeOH and (c) EtOH, within the 1D channels which follow the orientation indicated by the black arrow. Hydrogen bonds are marked as bicolour blue-red lines for N8⋯O1 and yellow-red for N2⋯O1. Atom colour code: Fe1 (blue octahedrons), Fe2 (orange octahedrons), N (blue), M (green), C (black). Note that one out of two possible positions found for the MeOH is shown.

The insertion of hydroxylic guest molecules in **1M** promotes different degrees of local distortion in the 2D framework, which are responsible for the formation of non-equivalent Fe^II^ and M^II^ (M^II^ = Pt, Pd) centres. The unsolvated **1M** derivatives, constituted of homogeneously corrugated 2D layers with a minimum degree of distortion (maximum symmetry), feature only one crystallographic Fe^II^ (and M^II^) site and show the occurrence of similar one-step complete cooperative SCO for M^II^ = Pt, Pd derivatives. In contrast, the inclusion of water distorts the layers generating two different centrosymmetric Fe^II^ sites in **1M·H2O** with different degrees of octahedral *Σ* and *Θ* distortions (see Table 2). The less distorted Fe1 site is more prone to exhibit SCO than that of Fe2 giving rise to the stabilization of an ordered intermediate mixed spin state ⋯LS(Fe1)–HS(Fe2)–LS(Fe1)⋯. The inclusion of MeOH or EtOH provokes further asymmetry in the **1M·0.5MeOH** and **1Pt·0.4EtOH** layers reflected on the occurrence of two crystallograpically different [M(CN)_4_]^2−^ centres and the loss of centrosymmetry in the Fe1 and Fe2 sites. Consequently, the SCO conversion occurs at lower temperatures involving essentially 50% of the Fe^II^ centres. Although the down-shift of the *T*_c_ parallels the increase of the guest size, the electronic factors may also play an important role (*vide infra*). Surprisingly, the Fe1 site, which undergoes SCO first in **1M·H2O**, remains HS in **1M·0.5MeOH**/**1Pt·0.4EtOH** even at 100 K, in spite of being surrounded by a less distorted octahedron (Table 2). However, the Fe2 site is SCO-active observing a complete HS → LS transition. In fact, pressure experiments carried out over **1Pt·0.5MeOH** demonstrate that whereas the *T*_c_ value of the Fe2 centre increases markedly with pressure, the pressure dependence of the SCO experienced by the Fe1 site is more moderate being almost complete only with pressures above 1.76 KBar (Fig. S13†). This singular situation can be associated with the fact that the oxygen atom of water and alcohol guests occupy different specific sites within the interlayer channels (Fig. 7). Indeed, the arrangement of methanol and ethanol molecules in the cavities tends to optimize the attractive interactions (H-bond) and minimize the repulsive contacts between the aliphatic part of the alcohol and the host network. Therefore, the water and the alcohol molecules display differences in the H-bond distances with the host 5-NH_2_Pym ligands (Table 1). More precisely, the methanol and ethanol molecules afford stronger H-bonds (shorter distances) than the water molecule with the non-coordinated N2 atom of the pyrimidine moiety, which is directly connected to the Fe1 sites. This fact explains the higher affinity to alcohols suggested by the adsorption isotherms, time-dependent TGAs and the hysteretic behaviour defined by their desorption isotherms. Since this H-bond withdraws electron density from the pyrimidine ring, it is reasonable to infer a decrease of the ligand field strength around the Fe1 sites “deactivating” the SCO. Furthermore, there are additional steric reasons involving contacts between the C atom(s) of the MeOH/EtOH and the pyrimidine ring coordinated to Fe1 [C(EtOH/MeOH)⋯C4(pym) and C(EtOH/MeOH)⋯N2(pym)] whose distances, shorter than the sum of the corresponding van der Waals radii, may also hamper the complete HS → LS transition stabilizing the mixed ⋯LS(Fe2)–HS(Fe1)–LS(Fe2)⋯ states. It is worthwhile emphasizing that the SCO behaviour of [Fe2N_6_] site remains mostly unaltered presenting very similar SCO temperatures when interacting with water or methanol, however, they decrease markedly with ethanol. In the case of **1Pd·0.25EtOH**, the low quantity of adsorbed ethanol seems to affect only a small fraction of Fe^II^ sites (*ca.* 32%) lowering their SCO temperature whereas the most part of the Fe^II^ sites (47%) exhibit SCO temperatures reminiscent of the **1Pd** unsolvated compound. This situation is reflected when observing the powder X-ray diffraction of **1Pd·0.25EtOH** soaked in ethanol, as the main intense peaks are those corresponding to the “empty” compound whereas only some less intense peaks correspond to the ethanol containing clathrate (Fig. S14†).

Finally, in order to qualitatively assess the selectivity properties of **1M** against the adsorption of H_2_O, MeOH and EtOH, freshly dehydrated **1M**·H_2_O samples were immersed overnight in solvent mixtures of H_2_O : MeOH, MeOH : EtOH or H_2_O : EtOH (1 : 1 in volume) and the SCO properties of the yielded solvates measured in the SQUID magnetometer. The results show that when soaking **1M** either in H_2_O : MeOH or MeOH : EtOH mixtures the recorded SCO curves are reminiscent of those of **1M·0.5MeOH** (Fig. S15†) indicating a higher tendency to adsorb MeOH over the other molecules. On the other hand, the magnetic properties of **1M**, recorded after being soaked in H_2_O : EtOH mixtures, display 2-stepped SCO profiles characteristic of **1M·H2O** derivatives (Fig. S15†) suggesting that water molecules have been mostly adsorbed in this case. These results are in good agreement with that expected from the adsorption isotherms and X-ray diffraction data which predict higher chemical affinity to MeOH than H_2_O and higher selectivity of H_2_O/MeOH against EtOH. The former observation may be associated to the stronger host–guest interactions stablished with MeOH whereas the latter can be interpreted as a molecular size-based exclusion in which the adsorption of the larger EtOH molecules is hampered by steric effects. Overall, the adsorption selectivity showed by **1M** follows the tendency MeOH > H_2_O > EtOH.

## Conclusions

In conclusion, SCSC transformations in the new family of 2D HCPs formulated {Fe(5-NH_2_Pym)_2_[M^II^(CN)_4_]}·G (**1M·G**, M = Pt or Pd, G = H_2_O, MeOH or EtOH) have revealed reversible and controllable guest-dependent structural transformations coupled to drastic SCO changes. The dual donor–acceptor nature of the 5-NH_2_Pym axial ligands affords a singular array of intra-layer H-bond interactions. In the guest-free **1M** derivative, these interactions involve 50% of the amino groups and non-coordinated N atoms of the 5-NH_2_Pym ligands coordinated to adjacent [FeN_6_] sites, thereby conferring strong undulation to the layered structure. The remaining 50% of NH_2_/N(pym) pairs, structurally disabled to define mutual H-bonds, generate functionalized void spaces potentially suitable for small ROH hydroxylic solvents. Indeed, exposition of the essentially non-porous **1M** derivatives to H_2_O, MeOH or EtOH induces a gate-opening adsorption mechanism which involves important structural reorganizations including 180° rotation of the 5-NH_2_Pym ligands, flattening of the layers and creation of host–guest H-bonds facilitating the migration of the trapped molecules. Importantly, the adsorption capabilities of **1M** depend not only on the nature of the guest molecule and that of the host framework (M = Pt or Pd) conferring selectivity properties to the system, but also on their reciprocal interactions. These mutual interactions in turn affect the Fe^II^ environments and determine varying and predictable SCO behaviours. The interplay between SCO and gate-opening adsorption, together with their likely suitability to be processed as nanometric thin films, as other related compounds,^21,27,30^ evidence the potential of the reported 2D amino-functionalized HCPs for sensing and/or gas separation applications.

## Conflicts of interest

There are no conflicts to declare.

## Supplementary Material

SC-011-D0SC04246C-s001

SC-011-D0SC04246C-s002

## References

[cit1] König E. (1991). Struct. Bond. (Berlin).

[cit2] Real J. A., Gaspar A. B., Muñoz M. C. (2005). Dalton Trans..

[cit3] Gütlich P., Ksenofontov V., Gaspar A. B. (2005). Coord. Chem. Rev..

[cit4] Decurtins S., Gütlich P., Köhler P. C., Spiering H., Hauser A. (1984). Chem. Phys. Lett..

[cit5] Lefter C., Tan R., Dugay J., Tricard S., Molnár G., Salmon L., Carrey J., Nicolazzi W., Rotaru A., Bousseksou A. (2016). Chem. Phys. Lett..

[cit6] Muñoz M. C., Real J. A. (2011). Coord. Chem. Rev..

[cit7] (b) Kumar K. S. and Ruben M., Angew. Chem., Int. Ed. 10.1002/ange.201911256

[cit8] Meneses-Sánchez M., Piñeiro-López L., Delgado T., Bartual-Murgui C., Muñoz M. C., Chakraborty P., Real J. A. (2020). J. Mater. Chem. C.

[cit9] Sato O., Li Z.-Y., Yao Z.-S., Kang S. and Kanegawa S., in Spin-Crossover Materials, John Wiley & Sons Ltd, 2013, pp. 303–319

[cit10] Piñeiro-López L., Seredyuk M., Muñoz M. C., Real J. A. (2020). Eur. J. Inorg. Chem..

[cit11] Real J. A., Andrés E., Muñoz M. C., Julve M., Granier T., Bousseksou A., Varret F. (1995). Science.

[cit12] Halder G. J., Kepert C. J., Moubaraki B., Murray K. S., Cashion J. D. (2002). Science.

[cit13] Garcia Y., Niel V., Munoz M. C., Real J. A. (2004). Top. Curr. Chem..

[cit14] Otsubo K., Haraguchi T., Kitagawa H. (2017). Coord. Chem. Rev..

[cit15] Ni Z.-P., Liu J.-L., Hoque Md. N., Liu W., Li J.-Y., Chen Y.-C., Tong M.-L. (2017). Coord. Chem. Rev..

[cit16] Ohba M., Yoneda K., Agustí G., Muñoz M. C., Gaspar A. B., Real J. A., Yamasaki M., Ando H., Nakao Y., Sakaki S., Kitagawa S. (2009). Angew. Chem., Int. Ed..

[cit17] Bartual-Murgui C., Akou A., Shepherd H. J., Molnar G., Real J. A., Salmon L., Bousseksou A. (2013). Chem.–Eur. J..

[cit18] Zenere K. A., Duyker S. G., Trzop E., Collet E., Chan B., Doheny P. W., Kepert C. J., Neville S. M. (2018). Chem. Sci..

[cit19] Liu F.-L., Tao J. (2017). Chem.–Eur. J..

[cit20] Liu W., Wang L., Su Y.-J., Chen Y.-C., Tucek J., Zboril R., Ni Z.-P., Tong M.-L. (2015). Inorg. Chem..

[cit21] Bartual-Murgui C., Rubio-Giménez V., Meneses-Sánchez M., Valverde-Muñoz F. J., Tatay S., Martí-Gastaldo C., Muñoz M. C., Real J. A. (2020). ACS Appl. Mater. Interfaces.

[cit22] Yang Q.-Y., Lama P., Sen S., Lusi M., Chen K.-J., Gao W.-Y., Shivanna M., Pham T., Hosono N., Kusaka S., Perry J. J., Ma S., Space B., Barbour L. J., Kitagawa S., Zaworotko M. J. (2018). Angew. Chem., Int. Ed..

[cit23] Horike S., Tanaka D., Nakagawa K., Kitagawa S. (2007). Chem. Commun..

[cit24] Avrami M. (1939). J. Chem. Phys..

[cit25] Nakaya M., Kosaka W., Miyasaka H., Komatsumaru Y., Kawaguchi S., Sugimoto K., Zhang Y., Nakamura M., Lindoy L. F., Hayami S. (2020). Angew. Chem., Int. Ed..

[cit26] Ohtani R., Shimayama K., Mishima A., Ohba M., Ishikawa R., Kawata S., Nakamura M., Lindoy L. F., Hayami S. (2015). J. Mater. Chem. C.

[cit27] Sakaida S., Otsubo K., Sakata O., Song C., Fujiwara A., Takata M., Kitagawa H. (2016). Nat. Chem..

[cit28] Barrios L. A., Bartual-Murgui C., Peyrecave-Lleixà E., Le Guennic B., Teat S. J., Roubeau O., Aromí G. (2016). Inorg. Chem..

[cit29] Coronado E., Giménez-Marqués M., Mínguez Espallargas G., Rey F., Vitórica-Yrezábal I. J. (2013). J. Am. Chem. Soc..

[cit30] Rubio-Giménez V., Bartual-Murgui C., Galbiati M., Núñez-López A., Castells-Gil J., Quinard B., Seneor P., Otero E., Ohresser P., Cantarero A., Coronado E., Real J. A., Mattana R., Tatay S., Martí-Gastaldo C. (2019). Chem. Sci..

