# Supplementary material for: Reversible guest-induced gate-opening with multiplex spin crossover responses in two-dimensional Hofmann clathrates
Source: Chem Sci. 2020 Sep 22;11(41):11224–34. doi: 10.1039/d0sc04246c (PMC8162911; doi:10.1039/d0sc04246c)

Electronic Supplementary Material (ESI) for Chemical Science.
This journal is © The Royal Society of Chemistry 2020

SUPPORTING INFORMATION

Reversible guest-induced gate-opening with multiplex spin crossover responses in two-dimensional Hofmann Clathrates

Rubén Turo-Cortés,[a] Carlos Bartual-Murgui,*[a] Javier Castells-Gil,[a] M. Carmen Muñoz,[b] Carlos Martí-Gastaldo,[a] and José A. Real*[a]

a. Departamento de Química Inorgánica, Instituto de Ciencia Molecular (ICMol), Universidad de Valencia, Valencia, Spain..

b. Departamento de Física Aplicada, Universitat Politècnica de València, Camino de Vera s/n, E-46022, Valencia, Spain.

Experimental section

Materials

All reagents and solvents used, including the 5-aminopyrimidine ligand, were obtained from commercial sources and used as received without further purification.

Synthetic procedure

Single crystals of {Fe(5-NH_2pmd)$_2$[Pt(CN)$_4$]}·H_2O and {Fe(5-NH_2pmd)$_2$[Pd(CN)$_4$]}·H_2O (**1^{Pt}·H_2O** and **1^{Pd}·H_2O**) were grown by slow liquid diffusion in a double H-shaped tube. A small amount of Fe(BF_4)$_2$·6H_2O (33.7 mg, 0.1 mmol) was deposited on one side, while the other positions were occupied by 5-aminopyrimidine (20.0 mg, 0.2 mmol) and K_2[Pd(CN)$_4$] or K_2[Pt(CN)$_4$] (34.1 mg or 43.1 mg, 0.1 mmol). The tube was filled with H_2O (ca. 10 mL). The tube was then sealed and left undisturbed for 4 weeks, after that time yellow plate single crystals appeared in 70% yield. Elemental Analysis: Calculated for **1^{Pt}·H_2O** [$C_{12}H_{12}FeN_{10}OPt$ (563.23) (%)]: C 24.87, H 2.15, N 24.87 Found (%): C 25.24, H 2.21, N 24.09; Calculated for **1^{Pd}·H_2O** [$C_{12}H_{12}FeN_{10}OPd$ (474.6) (%)]: C 30.37, H 2.55, N 29.52. Found (%): C 30.71, H 2.63, N 29.11.

1^{Pt} and **1^{Pd}** desolvated compounds were obtained by introducing crystals of **1^{Pt}·H_2O** and **1^{Pd}·H_2O** in an oven at 400K for 30 minutes. **1^{Pt}·0.5MeOH**, **1^{Pd}·0.5MeOH**, **1^{Pt}·0.4EtOH** and **1^{Pd}·0.25EtOH** were achieved by submerging freshly prepared **1^{Pt}** and **1^{Pd}** compounds in a bath of the corresponding solvent for ca. 3 hours. In all cases the compounds are yellow plate crystals at room temperature and turn red (for hydrated and dehydrated compounds) or orange (for alcohol containing

compounds) upon cooling at 100 K. Elemental Analysis: Calculated for **1^{Pt}·0.5MeOH** [$C_{12.5}H_{12}FeN_{10}O_{0.5}Pt$ (561.02) (%)]: C 26.75, H 2.16, N 24.96. Found (%): C 26.07, H 2.33, N 24.04; Calculated for **1^{Pd}·0.5MeOH** [$C_{12.5}H_{12}FeN_{10}O_{0.5}Pd$ (471.96) (%)]: C 31.77, H 2.56, N 29.64 Found (%): C 31.15, H 2.38, N 29.22; Calculated for **1^{Pt}·0.4EtOH** [$C_{12.8}H_{12.4}FeN_{10}O_{0.4}Pt$ (563.42) (%)]: C 27.28, H 2.22, N 24.85. Found (%): C 26.88, H 2.10, N 24.07; Calculated for **1^{Pd}·0.25EtOH** [$C_{12.4}H_{11.2}FeN_{10}O_{0.2}Pd$ (465.16) (%)]: C 31.98, H 2.42, N 30.07 Found (%): C 30.96, H 2.56, N 29.67.

Physical characterization

Elemental analyses (C, H, and N) were performed with a CE Instruments EA 1110 CHNS Elemental analyzer.

Magnetic measurements were performed with a Quantum Design MPMS-XL-5 SQUID magnetometer working in the 2 to 400 K temperature range with an applied magnetic field of 0.1 T. Experimental susceptibilities were corrected for diamagnetism of the constituent atoms by the use of Pascal's constants.

Calorimetric measurements were performed using a differential scanning calorimeter Mettler Toledo DSC 821e. Low temperatures were obtained with an aluminium block attached to the sample holder, refrigerated with a flow of liquid nitrogen and stabilized at a temperature of 110 K. The sample holder was kept in a dry box under a flow of dry nitrogen gas to avoid water condensation. The measurements were carried out using around 15 mg of polycrystalline samples sealed in aluminium pans with a mechanical crimp. Temperature and heat flow calibrations were made with standard samples of indium by using its melting transition (429.6 K, 28.45 J g^{-1}). An overall accuracy of ±0.2 K in temperature and ±2% in the heat capacity is estimated. The uncertainty increases for the determination of the anomalous enthalpy and entropy due to the subtraction of an unknown baseline.

Powder X-ray diffraction measurements where performed on a PANalytical Empyrean X-ray powder diffractometer (monochromatic CuKα radiation) in capillary measurement mode. Due to the spontaneous rehydration of **1^{Pt}** and **1^{Pd}**, these samples were prepared by heating the hydrated forms into open capillaries inside an oven at 120ºC during 1 hour and rapidly sealing them to avoid the entering of air.

Single crystal X-ray measurements. Single crystals were mounted on a glass fiber using a viscous hydrocarbon oil to coat the crystal and then transferred directly to the cold nitrogen stream for data collection. X-ray data were collected on a Supernova diffractometer equipped with a graphite-monochromated Enhance (Mo) X-ray Source (λ = 0.71073 Å). The program CrysAlisPro, Oxford Diffraction Ltd., was used for unit cell determinations and data reduction. Empirical absorption correction was performed using spherical harmonics, implemented in the SCALE3 ABSPACK scaling algorithm. The structures were solved by direct methods using SHELXS-2014 and refined by full matrix least-squares on F^2 using SHELXL-2014 (Sheldrick, G. M. Crystal Structure Refinement with SHELXL. Acta Crystallogr., Sect. C: Struct. Chem. 2015, 71, 3–8). Non-hydrogen atoms were refined anisotropically, and hydrogen atoms were placed in calculated positions refined using idealized geometries (riding model) and assigned fixed isotropic displacement parameters.

Adsorption/desorption isotherms. Vapor adsorption measurements were recorded on a Micromeritics 3Flex apparatus at relative pressures up to 1 bar and performed *ex situ* on $\mathbf{1}^{\mathbf{M}}$. Samples were degassed overnight at 150 ºC and 10^{-6} Torr prior to analysis assuring the presence of the totally desolvated $\mathbf{1}^{\mathbf{M}}$ compounds. A Micromeritics' ISO Controller was used to keep the temperature constant at 293 K for the H_2O, MeOH or EtOH adsorption measurements.

TGA experiments were carried out with a TA instruments TGA550 device equipped with a Pt/Rh oven (Tmax = 1000°C). The time dependent TGA experiments were performed by connecting the TGA apparatus to a flow mass controller. Thus, a controlled dry nitrogen flow (60 l/min) was passed through the desired solvent (water, methanol or ethanol) at room pressure and a temperature of 30°C and then the mixture (N_2+solvent vapor) was driven until the TGA chamber where a previously desolvated sample $\mathbf{1}^{\mathbf{M}}$ was mounted in a Pt pan.

Figure S1. Thermogravimetric analyses of a) $\mathbf{1^{Pt}{\cdot}H_2O}$, b) $\mathbf{1^{Pd}{\cdot}H_2O}$, c) $\mathbf{1^{Pt}{\cdot}0.5MeOH}$, d) $\mathbf{1^{Pd}{\cdot}0.5MeOH}$, e) $\mathbf{1^{Pt}{\cdot}0.4EtOH}$ and f) $\mathbf{1^{Pt}{\cdot}25EtOH}$.

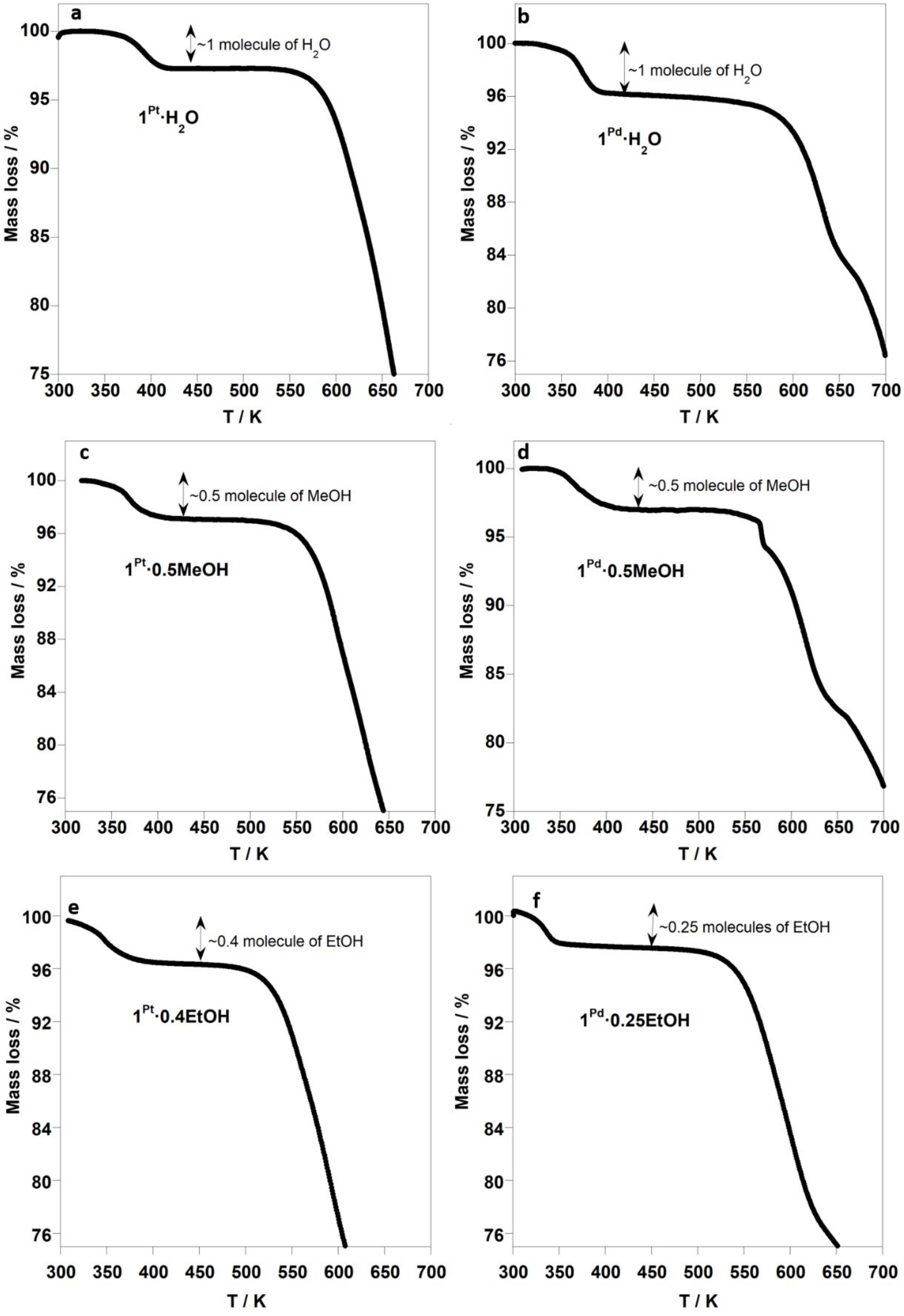

Figure S2. a) (001) and b) (100) views of three stacked {Fe(5-NH_2Pym)$_2$[M(CN)$_4$]} sheets in $\mathbf{1^M \cdot H_2O}$ (M = Pt or Pd). π-π interactions are highlighted with black dashed lines.

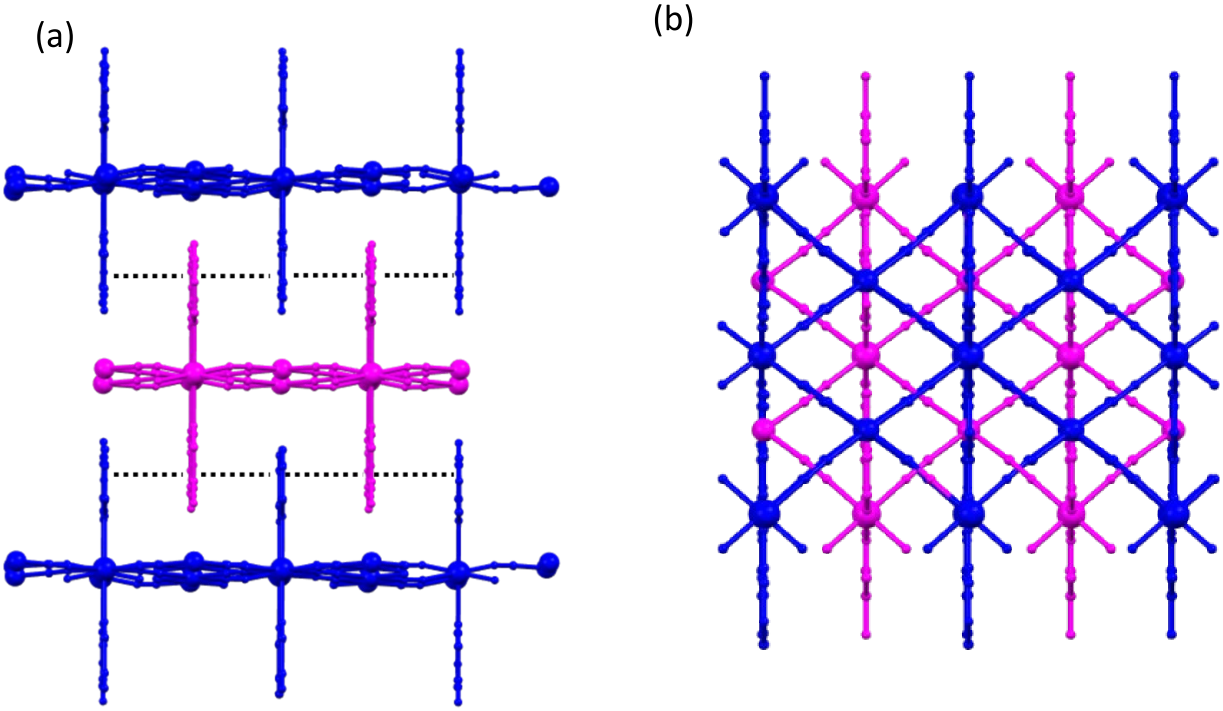

Table S1. Crystal data for $\mathbf{1^{Pt}{\cdot}H_2O}$ and $\mathbf{1^{Pt}}$ at the indicated temperatures.

	$\mathbf{1^{Pt}{\cdot}H_2O}$**_120K**	$\mathbf{1^{Pt}{\cdot}H_2O}$**_187K**	$\mathbf{1^{Pt}{\cdot}H_2O}$**_260K**	$\mathbf{1^{Pt}}$**_120K**	$\mathbf{1^{Pt}}$**_260K**
Empirical formula	$C_{12}H_{11.6}N_{10}O_{0.8}PtFe$	$C_{12}H_{11.6}N_{10}O_{0.8}PtFe$	$C_{12}H_{10}N_{10}O_{0.8}PtFe$	$C_{12}H_{10}N_{10}PtFe$	$C_{12}H_{10}N_{10}PtFe$
*M*r	559.65	559.65	558.04	545.24	545.24
Crystal system	monoclinic	monoclinic	monoclinic	orthorhombic	orthorhombic
Space group	*C2/m*	*C2/m*	*C2/m*	*Pnma*	*Pnma*
a (Å)	21.078(3)	21.488(3)	21.836(2)	14.1282(6)	14.5857(6)
b (Å)	7.0587(9)	7.1993(8)	7.3747(6)	7.1968(3)	7.3650(4)
c (Å)	14.488(3)	14.671(2)	14.8914(12)	15.7460(7)	15.9543(8)
β (°)	131.352(3)	131.584(3)	131.952(3)		
V (Å^3)	1618.1(5)	1697.6(3)	1783.4(3)	1601.02(12)	1713.87(14)
Z	4			4	
T (K)	120	187	260	120	260
D_c (mg cm^{-3})	2.297	2.190	2.078	2.262	2.113
F(000)	1056	1056	1050	1024	1024
μ (Mo-K$_\alpha$) (mm^{-1})	9.556	9.109	8.670	9.651	9.016
Crystal size (mm)	0.10x0.18x0.18				
No. of total reflections	3046	2871	3361	2821	2164
No. of reflections [$I>2\sigma(I)$]	2106	2407	2601	2240	1596
R [$I>2\sigma(I)$]	0.0814	0.0737	0.0544	0.0470	0.0520
wR [$I>2\sigma(I)$]	0.1954	0.1674	0.1267	0.1059	0.1297
S	1.108	1.139	1.036	1.124	1.190

$R = \Sigma ||Fo| - |Fc|| / \Sigma |Fo|$; $wR = [\Sigma [w(Fo^2 - Fc^2)^2] / \Sigma [w(Fo^2)^2]]^{1/2}$.
$w = 1/ [\sigma^2(Fo^2) + (m\,P)^2 + n\,P]$ where $P = (Fo^2 + 2Fc^2) / 3$;
m = 0.1197 (**1**), 0.0265 (**2**), 0.0685 (**3**), 0.0000 (**4**), and 0.0523 (**5**);
n = 97.5723 (**1**), 170.7754 (**2**), 41.0043 (**3**), 52.1055 (**4**), and 43.7598 (**5**)

Table S2. Crystal data for $\mathbf{1^{Pd}{\cdot}H_2O}$ and $\mathbf{1^{Pd}}$ at the indicated temperatures.

	$\mathbf{1^{Pd}{\cdot}H_2O}$**_120K**	$\mathbf{1^{Pd}{\cdot}H_2O}$**_180K**	$\mathbf{1^{Pd}{\cdot}H_2O}$**_260K**	$\mathbf{1^{Pd}}$**_120K**	$\mathbf{1^{Pd}}$ **_260K**
Empirical formula	$C_{12}H_{12}N_{10}OPdFe$	$C_{12}H_{12}N_{10}OPdFe$	$C_{12}H_{10}N_{10}OPdFe$	$C_{12}H_{10}N_{10}PdFe$	$C_{12}H_{10}N_{10}PdFe$
*M*r	474.57	474.57	472.55	456.55	456.55
Crystal system	monoclinic	monoclinic	monoclinic	orthorhombic	orthorhombic
Space group	*C2/m*	*C2/m*	*C2/m*	*Pnma*	*Pnma*
a (Å)	21.092(3)	21.454(3)	21.770(3)	14.1549(7)	14.2723(10)
b (Å)	7.0553(10)	7.1986(8)	7.3679(8)	7.1808(4)	7.3843(5)
c (Å)	14.535(2)	14.7221(16)	14.951(3)	15.6363(8)	15.9189(12)
β (°)	131.372(4)	131.494(3)	131.902(3)		
V (Å^3)	1623.1(4)	1703.0(3)	1784.9(5)	1589.33(14)	1677.7(2)
Z	4			4	
T (K)	120	180	260	120	260
D_c (mg cm^{-3})	1.942	1.851	1.758	1.908	1.808
F(000)	936	936	928	896	896
μ (Mo-K$_\alpha$) (mm^{-1})	2.027	1.932	1.843	2.061	1.953
Crystal size (mm)	0.05x0.15x0.20				
No. of total reflections	2266	2496	2671	2347	2021
No. of reflections [$I>2\sigma(I)$]	1699	1942	2122	1759	1419
R [$I>2\sigma(I)$]	0.0654	0.0564	0.0526	0.0644	0.0902
wR [$I>2\sigma(I)$]	0.1332	0.1276	0.1292	0.1465	0.2291
S	1.042	1.051	1.104	1.117	1.176

$R = \Sigma ||Fo| - |Fc|| / \Sigma |Fo|$; $wR = [\Sigma [w(Fo^2 - Fc^2)^2] / \Sigma [w(Fo^2)^2]]^{1/2}$.
$w = 1/ [\sigma^2(Fo^2) + (m\,P)^2 + n\,P]$ where $P = (Fo^2 + 2Fc^2) / 3$;
m = 0.0176 (**1**), 0.0445 (**2**), 0.0674 (**3**), 0.0457 (**4**), and 0.0403 (**5**);
n = 78.1367 (**1**), 42.1677 (**2**), 24.3621 (**3**), 18.8066 (**4**), and 73.0831 (**5**)

Figure S3. Powder X-ray diffraction patterns of a) $\mathbf{1^M \cdot H_2O}$, b) $\mathbf{1^M \cdot MeOH}$ and $\mathbf{1^M \cdot EtOH}$ and c) $\mathbf{1^M}$ series. Simulated patterns are also displayed for comparison.

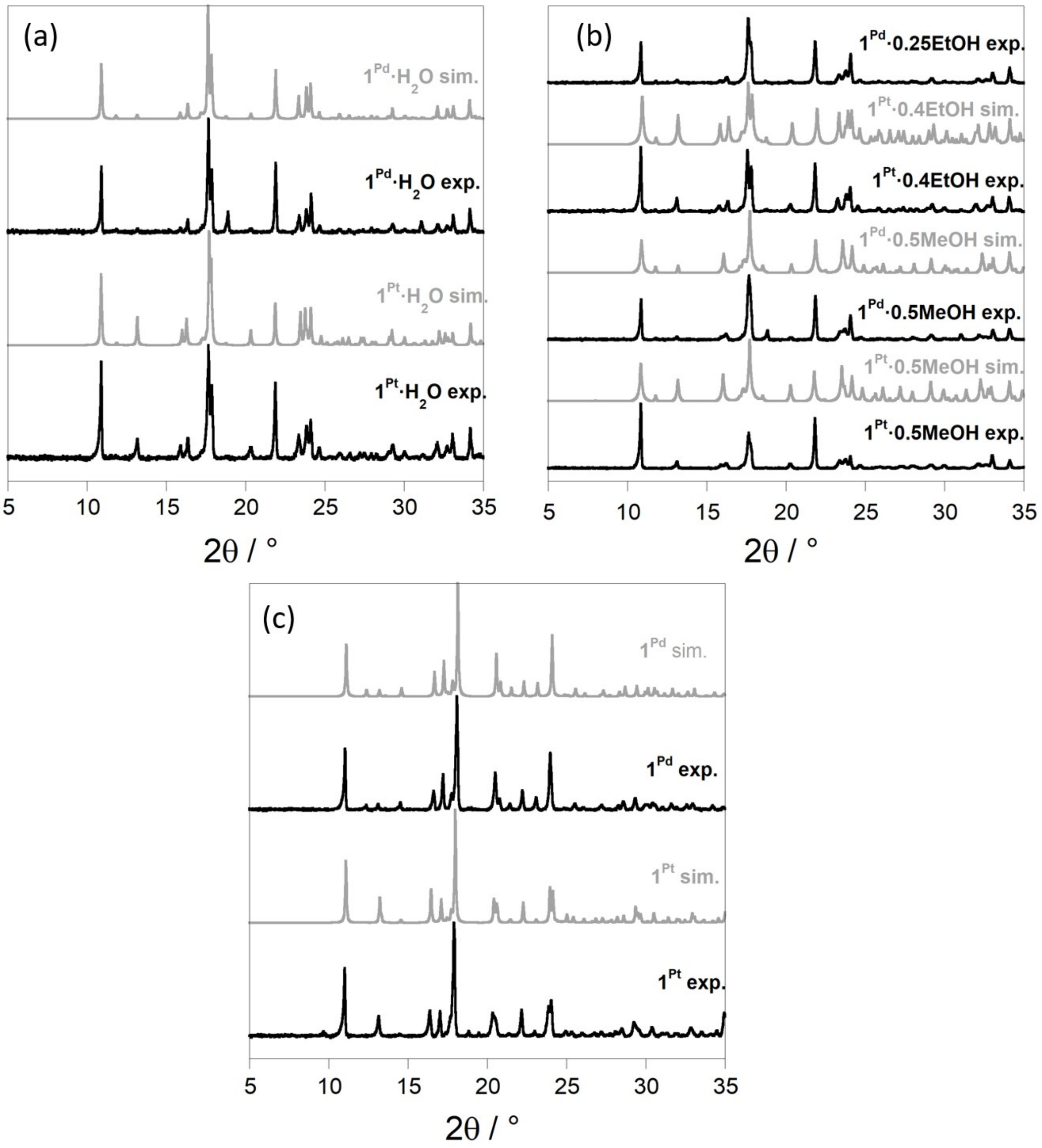

Time dependent TGA measurements

Kinetic water, methanol and ethanol adsorption experiments were performed through TGA measurements for activated **1**^{Pt} and **1**^{Pd} compounds (Figure S2a and S2b). As observed in Figure 2a, the tetracyanoplatinate derivative adsorbs 0.97/0.41/0.14 molecules of water/methanol/ethanol per Fe^{II} ion. However, in the same conditions, the tetracyanopalladate network adsorbs a smaller fraction of guest molecules (0.91/0.25/0.01 molecules of water/methanol/ethanol per Fe^{II} ion) during the same range of time. In order to estimate the adsorption rates exhibited by each derivative for the different guests, the experimental isotherm curves were fitted with the kinetic Avrami equation:

$$\alpha = A(1 - exp\left\{ - K_{av}t^{n} \right\})$$

where α is the adsorbed fraction at time t, A is the total amount of adsorbed guest at time ∞, K_{av} is the rate constant, and n is the Avrami exponent which defines the cooperativity of the adsorption process. The kinetic parameters resulting from the fittings are gathered in Table S1. Whereas methanol adsorption curves show higher uptake rates, those of water exhibit higher n values reflecting a certain degree of cooperativity throughout the adsorption process. Besides, for a given guest, **1**^{Pt} displays higher kinetic constants and lower n values than **1**^{Pd}.

Figure S4. Time dependent TGA measurements registered during the adsorption of water, methanol and ethanol at room temperature and pressure. Solid lines correspond to the corresponding fittings following the Avrami's equation.

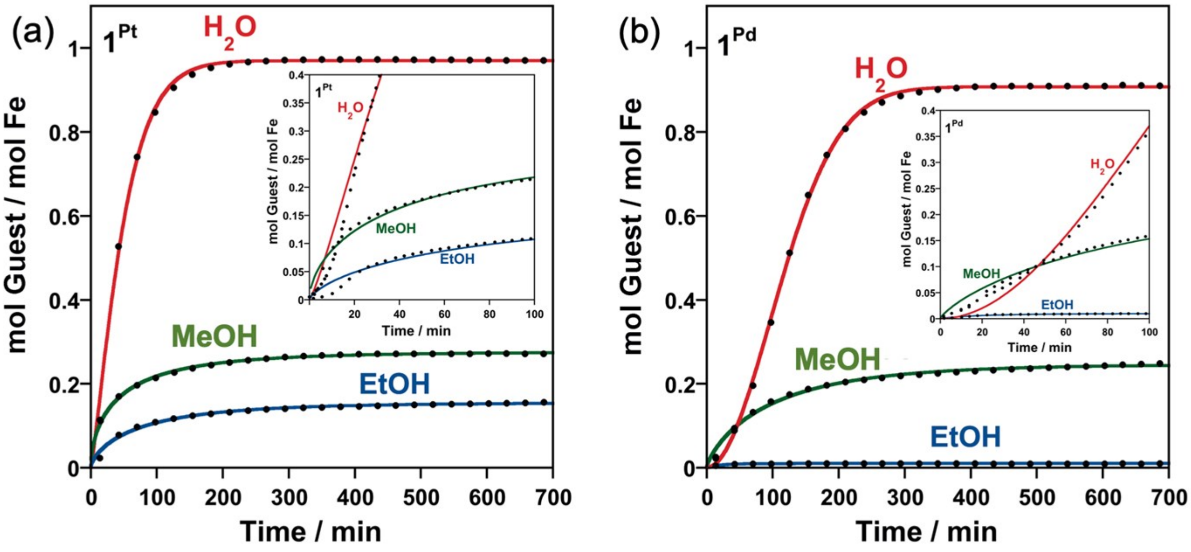

Table S3. Avrami parameters extracted from fitting of the experimental TGA curves depicted in Figure 4. **1^{Pd}·EtOH** has been excluded from fitting because it does not present significant adsorption at the studied conditions.

	K_{av}	*n*	*A*
**1^{Pt}+H_2O**	$7.00x10^{-3}$	1.25	0.97
**1^{Pd}+H_2O**	$6.35x10^{-5}$	1.96	0.91
**1^{Pt}+MeOH**	$9.38x10^{-2}$	0.61	0.27
**1^{Pd}+MeOH**	$2.43x10^{-2}$	0.80	0.25
**1^{Pt}+EtOH**	$4.45x10^{-2}$	0.71	0.15

Table S4. Crystal data for **1^{Pt}·0.5MeOH** and **1^{Pt}·0.4EtOH** at the indicated temperatures.

	**1^{Pt}·0.5MeOH _100K**	**1^{Pt}·0.5MeOH_260K**	**1^{Pt}·0.4EtOH_100K**	**1^{Pt}·0.4EtOH _260K**
Empirical formula	$C_{12.5}H_{12}N_{10}O_{0.5}PtFe$	$C_{12.5}H_{12}N_{10}O_{0.5}PtFe$	$C_{12.8}H_{10}N_{10}O_{0.4}PtFe$	$C_{12.8}H_{10}N_{10}O_{0.4}PtFe$
*M*r	561.26	561.26	561.25	561.25
Crystal system	orthorhombic		monoclinic	monoclinic
Space group	*Pbcm*		*I*2/*m*	*I*2/*m*
a (Å)	14.8631(7)	14.9943(8)	14.848(2)	14.980(2)
b (Å)	32.144(2)	32.626(2)	7.1717(7)	7.3662(6)
c (Å)	7.1639(4)	7.3615(3)	15.880(3)	16.181(2)
β (°)			92.699(11)	91.922(9)
V ($Å^3$)	3422.6(3)	3601.3(3)	1689.2(4)	1784.5(3)
Z	8	8	4	4
T (K)	100	260	100	260
D_c (mg cm^{-3})	2.178	2.070	2.207	2.089
F(000)	2120	2118	1056	1056
μ (Mo-K_α) (mm^{-1})	9.035	8.587	9.153	8.664
Crystal size (mm)	0.02x0.10x0.10	0.02x0.10x0.10	0.02x0.15x0.15	0.02x0.15x0.15
No. of total reflections	3804	4199	2079	2191
No. of reflections [$I>2\sigma(I)$]	3032	2751	1857	1902
R [$I>2\sigma(I)$]	0.0979	0.0869	0.1183	0.0693
wR [$I>2\sigma(I)$]	0.1933	0.1624	0.2800	0.1773
S	1.084	1.056	1.194	1.090

$R = \Sigma ||Fo| - |Fc|| / \Sigma |Fo|$; $wR = [\Sigma [w(Fo^2 - Fc^2)^2] / \Sigma [w(Fo^2)^2]]^{1/2}$.
$w = 1/ [\sigma^2(Fo^2) + (m P)^2 + n P]$ where $P = (Fo^2 + 2Fc^2) / 3$;
m = 0.0000 (**1**), 0.0333 (**2**), 0.1097 (**3**), and 0.1006 (**4**);
n = 553.5499 (**1**), 253.2535 (**2**), 251.023 (**3**), and 63.5736 (**4**)

Table S5. Crystal data for $\mathbf{1^{Pt}}$**·0.5MeOH** at the indicated temperatures.

	**FePd0.5(CH4O)_100K**	**FePd0.5(CH4O)_260K**
Empirical formula	$C_{12.5}H_{12}N_{10}O_{0.5}PdFe$	$C_{12.5}H_{12}N_{10}O_{0.5}PdFe$
*M*r	472.57	472.57
Crystal system	orthorhombic	
Space group	*Pbcm*	
a (Å)	14.9105(14)	15.0184(12)
b (Å)	32.021(2)	32.516(2)
c (Å)	7.1610(4)	7.3514(4)
V (Å^3)	3419.0(4)	3589.9(4)
Z	8	8
T (K)	120	260
D_c (mg cm^{-3})	1.836	1.749
F(000)	1864	1864
μ (Mo-K$_\alpha$) (mm^{-1})	1.922	1.830
Crystal size (mm)	0.03x0.12x0.12	0.03x0.12x0.12
No. of total reflections	3757	3897
No. of reflections [I>2σ(I)]	2486	1884
R [I>2σ(I)]	0.1088	0.0816
wR [I>2σ(I)]	0.2488	0.2037
S	1.041	1.050

$R = \Sigma \,||Fo| - |Fc|| \,/\, \Sigma\, |Fo|$; $wR = [\,\Sigma\,[w(Fo^2 - Fc^2)^2] \,/\, \Sigma\,[w(Fo^2)^2]]^{1/2}$.
$w = 1/\,[\sigma^2(Fo^2) + (\mathrm{m}\,P)^2 + \mathrm{n}\,P]$ where $P = (Fo^2 + 2Fc^2)\,/\,3$;
m = 0.0809 (**1**), and 0.0848 (**2**);
n = 178.4426 (**1**), and 25.2612 (**2**)

Figure S5. X-ray diffraction pattern evolution indicating the structural reversibility throughout the $\mathbf{1^M \cdot H_2O \rightarrow 1^M \rightarrow 1^M \cdot H_2O}$ process [M = Pt (left) or Pd (right)]. The dehydration and rehydration processes were carried out through a thermal treatment at 400 K during 30 minutes and by exposing the sample to air, respectively.

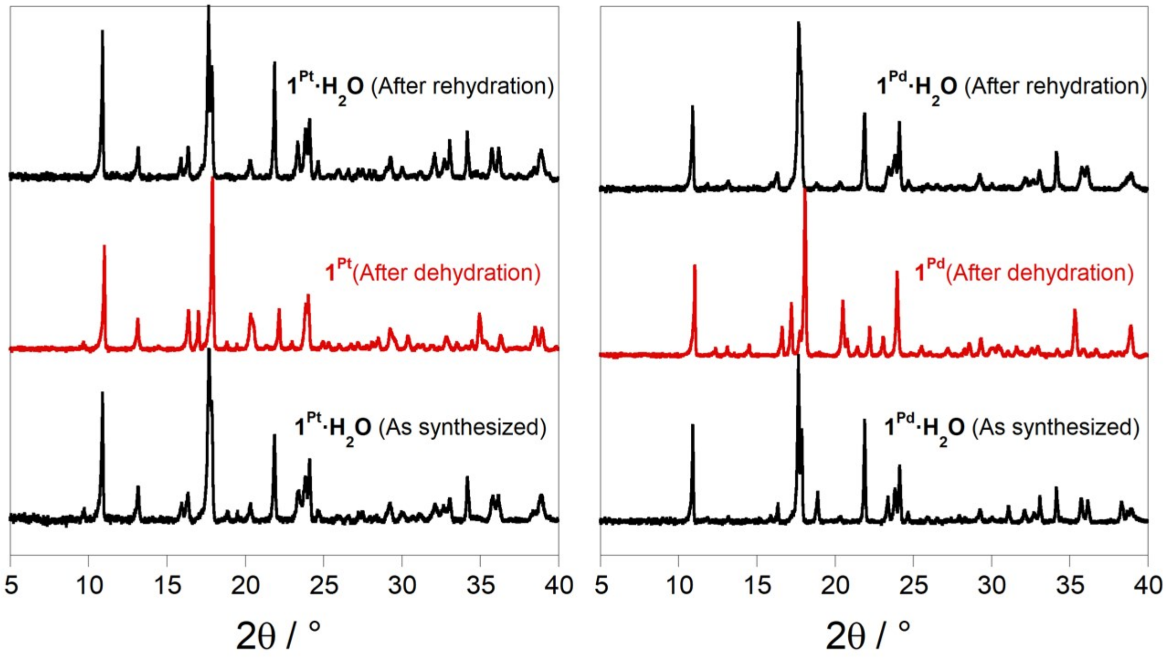

Figure S6. Asymmetric unit of **1^{Pt}·0.5MeOH** (isostructural to **1^{Pd}·0.5MeOH**). Hydrogen atoms have

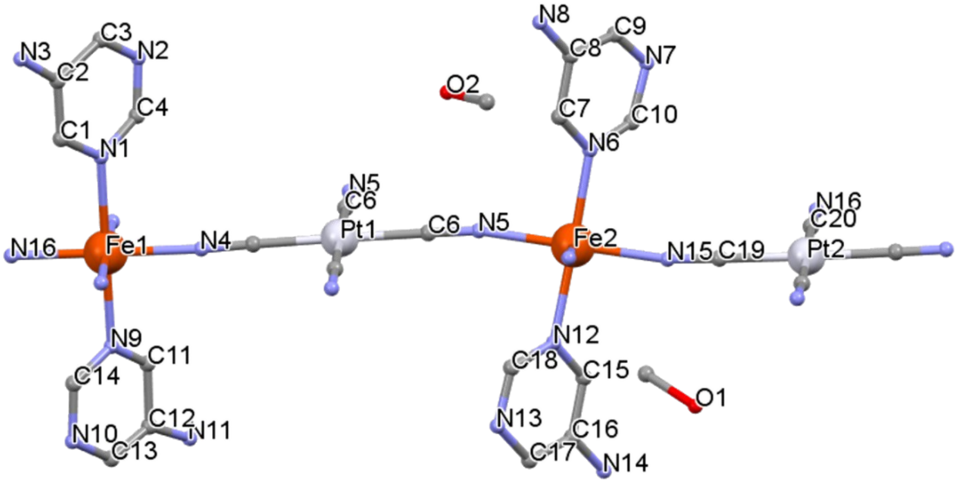

been omitted for clarity.

Figure S7. Fragment of the **1^{M}·0.5MeOH** structure displaying the packing of the {Fe[M(CN)$_4$]} layers and the 1D channels where the methanol guests are located. The N7···N3 and N11···N13 intralayer H-bonds are represented by black dashed lines.Figure S4. SCO properties of **1^{M}·H_2O** (M = (a) Pt or

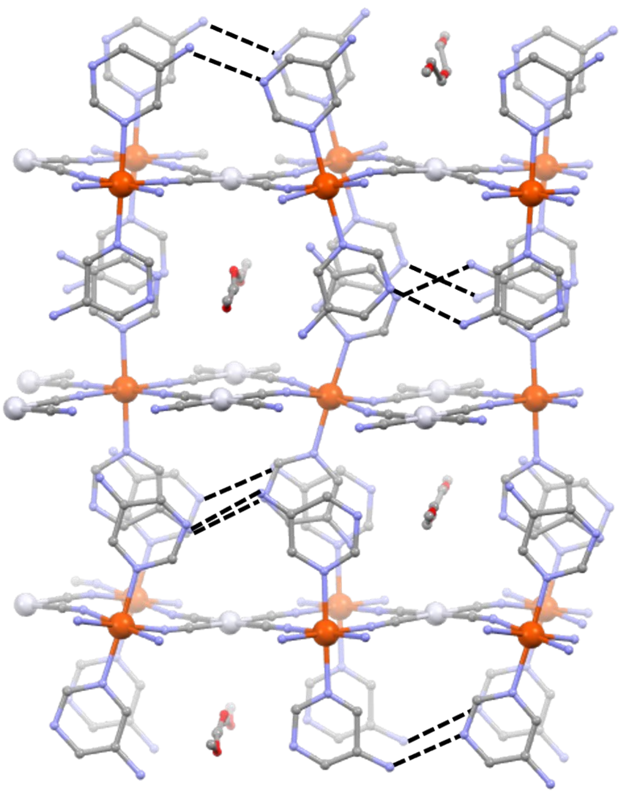

(b) Pd) before heating (left), after heating at 400 K for 30 minutes (middle) and after exposure of **1**^{Pt} to air for 3 hours (right).

Figure S8. X-ray diffraction pattern evolution during the **1**^{M}**·H**$_2$**O** $\rightarrow$ **1**^{M} $\rightarrow$ **1**^{M}**·0.5MeOH** $\rightarrow$ **1**^{M} guest exchange processes for M = a) Pt or b) Pd.

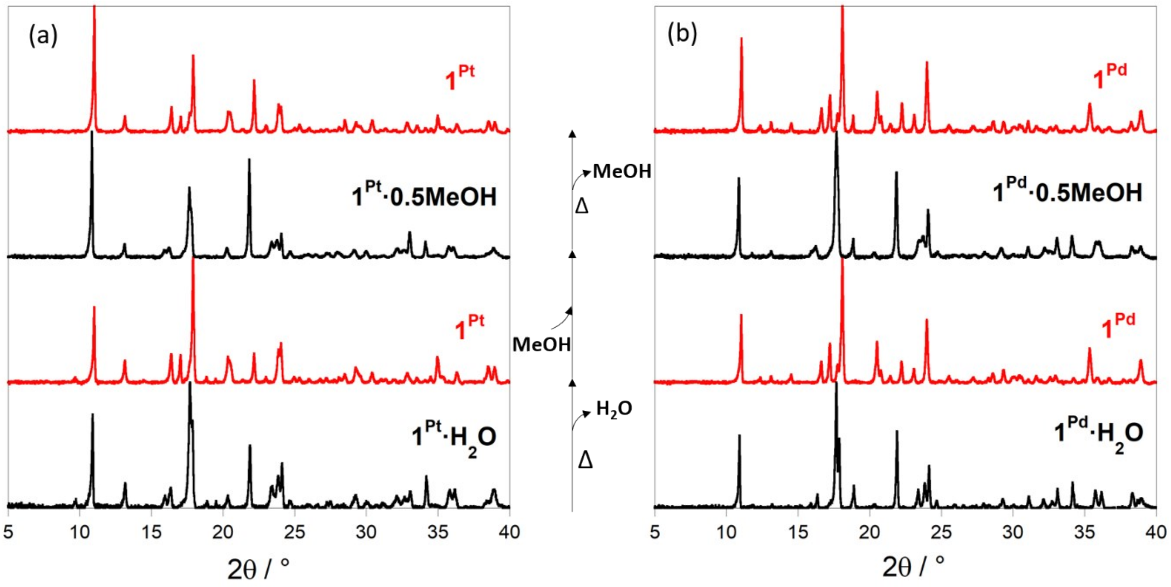

Figure S9. SCO properties recorded at 2 K min^{-1} of $\mathbf{1^{M}\cdot H_2O}$ (M = (a) Pt or (b) Pd) before heating (left), after heating at 400 K for 30 minutes (middle) and after exposure of $\mathbf{1^{Pt}}$ to air for 3 hours (right).

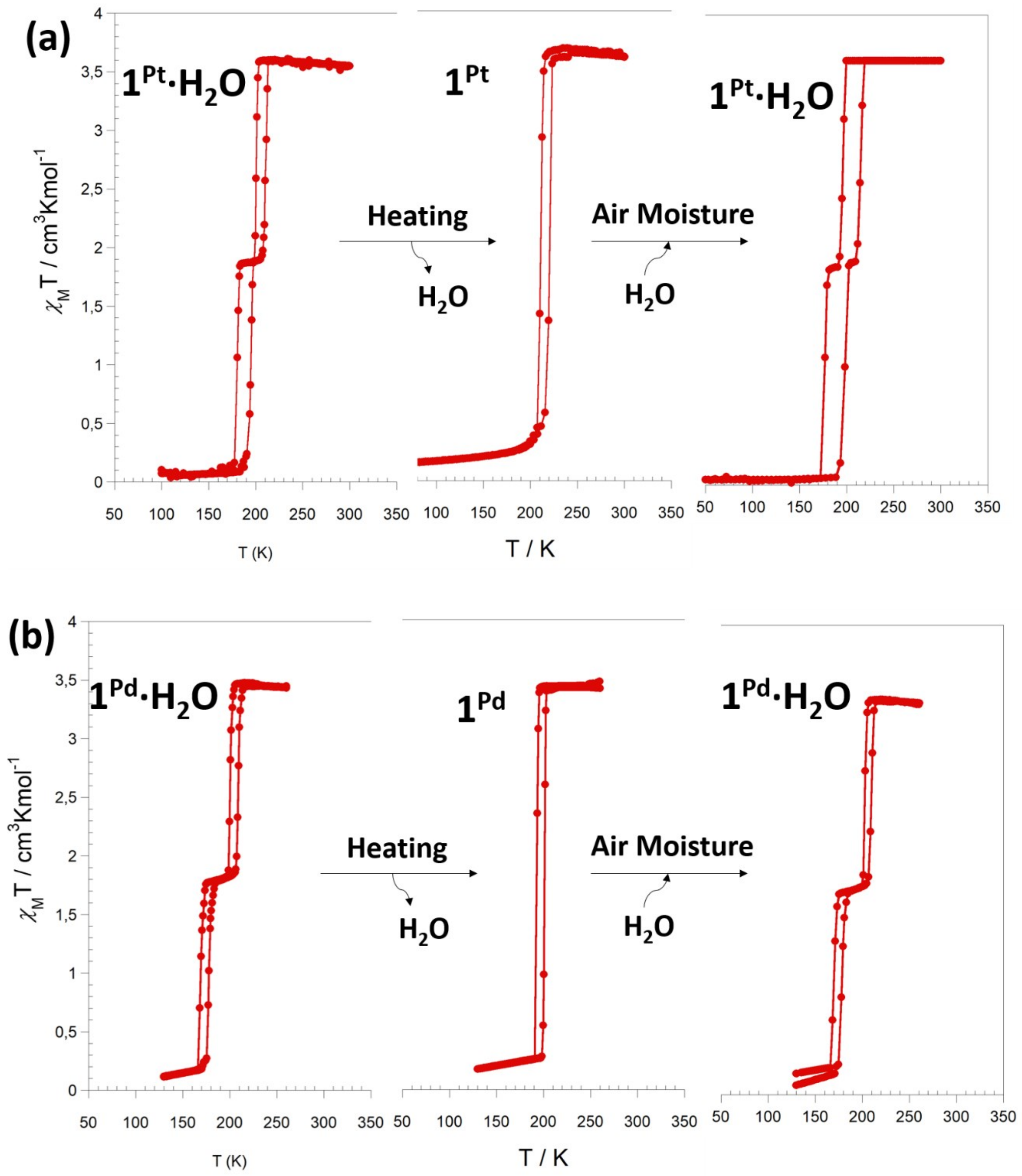

Figure S10. a) χ_MT *vs* T curves measured at 2 K min^{-1} of **1^{Pt}·0.4EtOH**, **1^{Pd}·0.5MeOH** and **1^{Pt}·0.5MeOH** in the 50-250 K range.

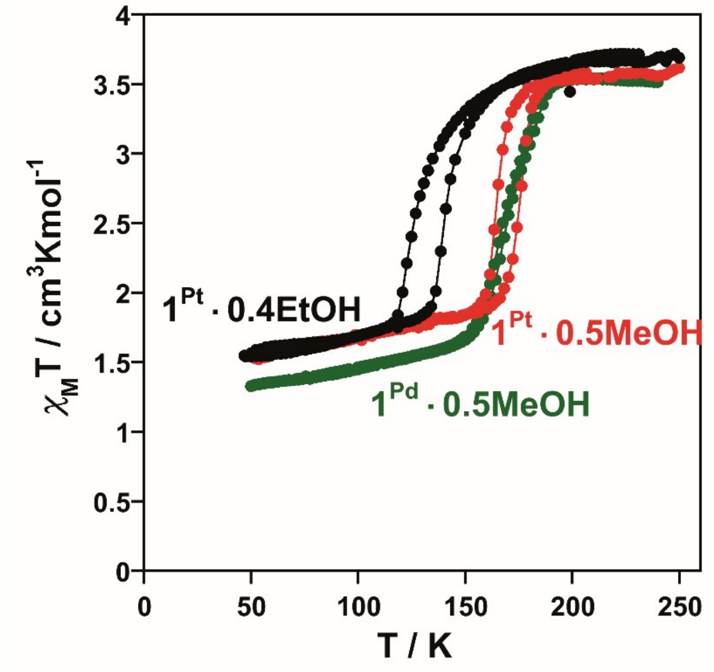

Figure S11. a) χ_MT *vs* T curve measured at 2 K min^{-1} of **1^{Pd}·0.25EtOH** (χ_MT *vs* T curves of **1^{Pt}·0.4EtOH** and **1^{Pd}** are also represented for comparison) and b) corresponding TGA analyses of **1^{Pd}·0.25EtOH** and **1^{Pt}·EtOH** revealing the presence of 0.25 and 0.42 molecules of EtOH per Fe^{II} atom, respectively.

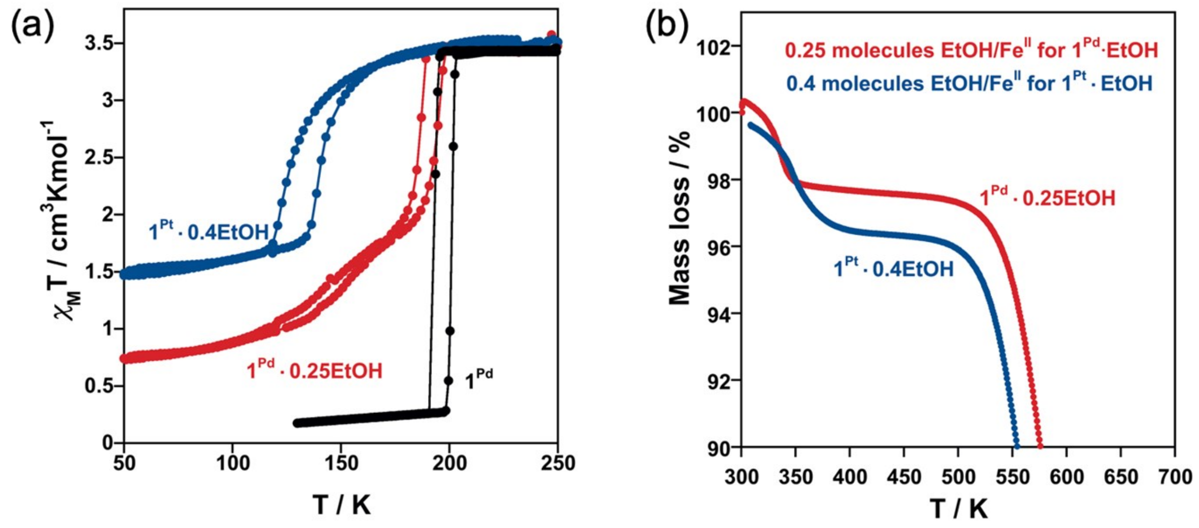

Figure S12. Calorimetric properties of $\mathbf{1^M}$, $\mathbf{1^M{\cdot}H_2O}$ and $\mathbf{1^M{\cdot}0.5MeOH}$ (M = Pt, Pd). Blue and red dashed lines correspond to the cooling and heating modes, respectively. The SCO curve of each derivative is included as a reference (grey lines).

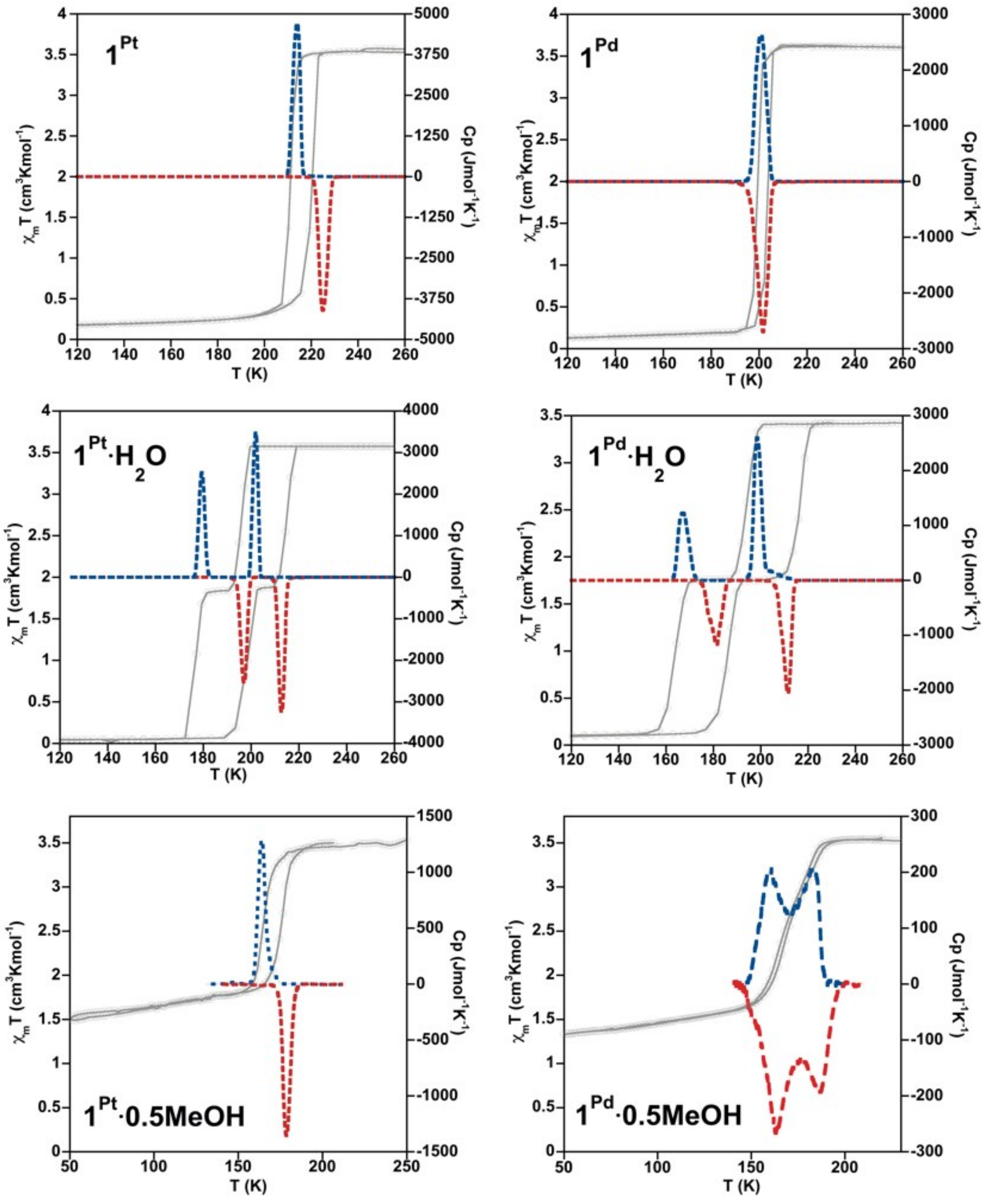

Figure S13. χT *vs* T plots for **1^{Pt}·0.5MeOH** at increasing pressures (scan rate: 2 K min^{-1}).

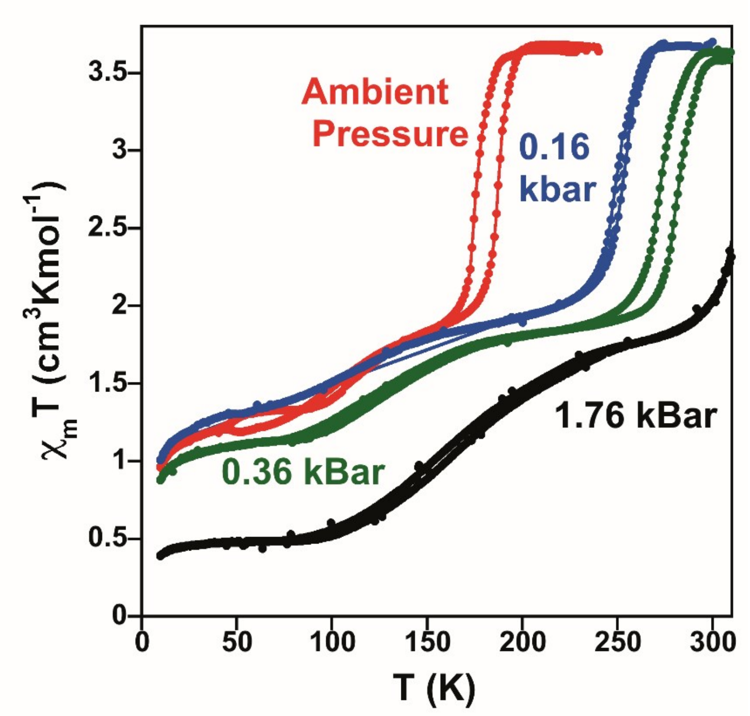

Table S6. Calculated accessible void volume (in Å^3) of the different clathrates for the different spin states.

	1^M		$1^M \cdot H_2O$			$1^M \cdot 0.5MeOH$		$1^M \cdot xEtOH$	
	HS	LS	HS-HS	HS-LS	LS-LS	HS-HS	HS-LS	HS-HS	HS-LS
M = Pt	No AVV observed		164	143	117	360	305	174	144
M = Pd			150	139	123	336	301		

Figure S14. Powder X-ray diffraction pattern of **1^{Pd}·EtOH** soaked in ethanol (blue line). Diffraction patterns of **1^{Pd}** (red line) and **1^{Pt}·EtOH** are also showed for comparison. Three insets corresponding to the 2θ ranges delimited by the red dashed areas are also depicted to see the spectra in more detail. As explained in the main text, the pattern of **1^{Pd}·EtOH** is very similar to that of **1^{Pd}** indicating that a low quantity of EOH is adsorbed by **1^{Pd}** and consequently the spectrum is barely modified. However, the inset plots reveal some weak peaks (indicated by black arrows) which are reminiscent of the **1^{Pd}·EtOH**.

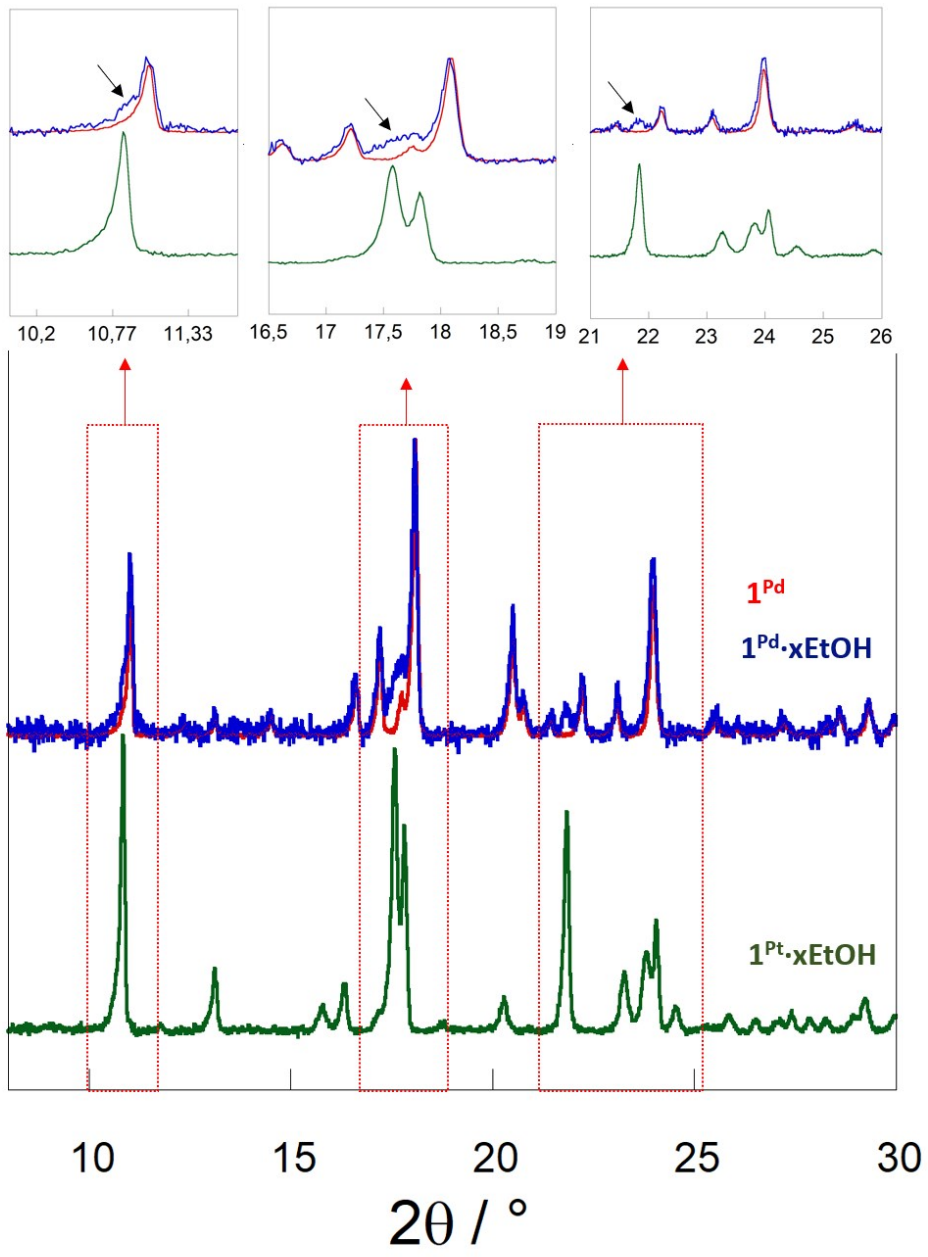

Figure S15. $\chi_M T$ *vs* T curves measured at 2 K min^{-1} for freshly dehydrated (a) **1^{Pt}** or (b) **1^{Pd}** compounds soaked in MeOH:EtOH, H_2O:MeOH or H_2O:EtOH (1:1) solvent mixtures. The curves of **$1^{M}\cdot H_2O$**, **$1^{M}\cdot 0.5MeOH$** (M = Pt, Pd) and **$1^{Pt}\cdot 0.4EtOH$** compounds are displayed in grey for comparison.

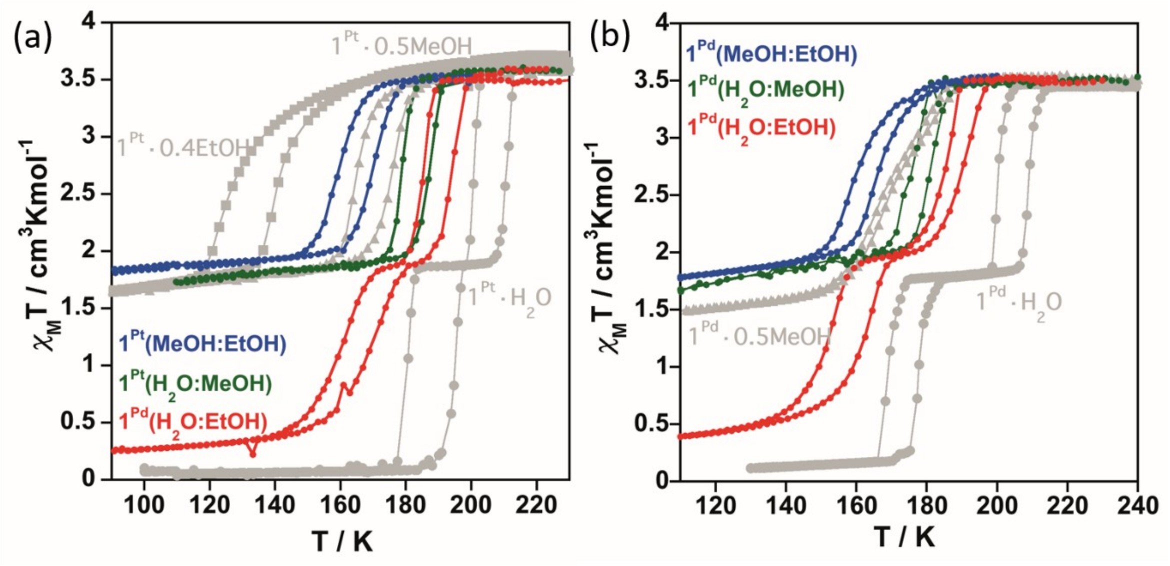

Supplement: SC-011-D0SC04246C-s001 [file SC-011-D0SC04246C-s001.pdf]
